# Effect of recombination on genetic diversity of *Caenorhabditis elegans*

**DOI:** 10.1038/s41598-023-42600-5

**Published:** 2023-09-30

**Authors:** Ho-Yon Hwang, Jiou Wang

**Affiliations:** Department of Biochemistry and Molecular Biology, Bloomberg School of Public Health, Department of Neuroscience, School of Medicine, Johns Hopkins University, Baltimore, MD 21205 USA

**Keywords:** Molecular evolution, Phylogenetics

## Abstract

Greater molecular divergence and genetic diversity are present in regions of high recombination in many species. Studies describing the correlation between variant abundance and recombination rate have long focused on recombination in the context of linked selection models, whereby interference between linked sites under positive or negative selection reduces genetic diversity in regions of low recombination. Here, we show that indels, especially those of intermediate sizes, are enriched relative to single nucleotide polymorphisms in regions of high recombination in *C. elegans*. To explain this phenomenon, we reintroduce an alternative model that emphasizes the mutagenic effect of recombination. To extend the analysis, we examine the variants with a phylogenetic context and discuss how different models could be examined together. The number of variants generated by recombination in natural populations could be substantial including possibly the majority of some indel subtypes. Our work highlights the potential importance of a mutagenic effect of recombination, which could have a significant role in the shaping of natural genetic diversity.

## Introduction

Variant abundance could be assessed by the number of DNA variants in different DNA intervals. In an individual, one interval could have more variants than another. Populations could have different numbers of haplotypes in different intervals, and more haplotypes mean more genetic diversity and more variants. Correlation between variant abundance and recombination rate is a feature in natural populations of many species including humans, mice, fruit flies, and nematodes^1–4^. Linked selection models have been used to explain this correlation^5^. One model is selective sweep, which is a consequence of genetic hitchhiking involving fixation of neutral variants that are closely linked to a beneficial mutation^6^. Another is background selection, which could cause extinction of neutral variants that are closely linked to a deleterious mutation^7^. Both selective sweep and background selection have a stronger effect in removing variants in regions of low recombination. Background selection is more important in species with a high inbreeding rate, such as *C. elegans*, with linked effects including low effective population size and linkage disequilibrium^8^. Between selective sweep and background selection, only selective sweep could reduce diversity and remove haplotypes near the site of selection^9^. Beyond linked selection models, accumulation of mutations could lead to more variants in the regions of high recombination according to a mutation model^10^.

Recombination has long been known to generate mutations, such as chromosomal rearrangements stemming from unequal crossover of chromosomes^11^. More recently, the mutagenic effect of chromosomal crossovers in generating de novo mutations was demonstrated examining human sperm^12^ and many human parents and offspring^13^. Chromosomal crossover is preceded by programmed DNA double-strand breaks, which is catalyzed by Spo11 topoisomerase-like protein^14, 15^. Meiotic double strand breaks are not always accompanied by chromosomal crossover but need to be repaired, which involve many genetic pathways^16^. Repair of DNA damage risks error, and thus double strand breaks could lead to mutation without directly involving crossover and recombination and thereby increase the number of mutations linked to recombination in the mutation model.

The mutation model was introduced previously^10^ to explain different correlations between recombination rate and different variant subtype proportions in natural variants identified by the Million Mutation Project (MMP)^17^. To confirm that these differences are real, we assembled new variant data using newer and better variant callers examining available long-read and short-read sequence data including those from Caenorhabditis elegans Natural Diversity Resource (CeNDR)^18, 19^. The new data sets also show differences in the strength of association between recombination rate and many variant subtype abundance. Different strengths of associations parallel the differences in the correlations between recombination rate and the proportions of various variant subtypes. Here, we provide refinements to the analysis of variant data with the mutation model, and we use phylogenetic examination to separate the effect of selective sweep from mutation and background selection.

## Results

### Strong correlation exists between recombination rate and abundance and proportion of indels

Whole-genome sequence data of many *C. elegans* wild isolates now exist. These include Illumina paired-end data of over 600 wild isolates by CeNDR, which also obtained first-generation PacBio long-read data of 14 wild isolates. Second-generation PacBio HiFi data^20^ and Oxford Nanopore Technologies (ONT) long-read data^21^ are available with CB4856 HA from Hawaii, which is the most intensively studied *C. elegans* wild isolate aside from the standard wild type strain N2. Illumina, PacBio, and ONT data is available with an N2-derivative with no known history of mutagenesis called VC2010 or PD1074^22, 23^ and is called CGC1 at Caenorhabditis elegans genetics center. Newer variant calling methods are also now available. Here, we evaluated Clair3^24^, BCFtools mpileup^25^, GATK HaplotypeCaller^26^, and DeepVariant^27^ for SNP and short indel calling, and we evaluated Sniffles2^28, 29^, SVIM^30^, and Dysgu^31^ for bigger indel calling.

Assessment of quality of variant calling was made primarily using CB4856 HA data of Illumina, ONT, and PacBio HiFi. Visual examinations using Integrated Genome Viewer (IGV)^32^ were made of sequence alignments prepared with Minimap2^33, 34^ and SAMtools^35^. Variants called by only a single variant caller are often dubious. Of the SNP and short indel callers, we deem Clair3 to be the most accurate because most variants called by Clair3 but not other callers looked quite good. With indel calling using long-read data, Sniffles2 has by far the best false positive and false negative rates, and indel call quality is marginally better with ONT as compared to PacBio HiFi and considerably worse with first-generation PacBio data. Many indel calls have poor precision with long-read data, and many false positives exist among SNP and small indel with long-read data. Summarizing our evaluation, which is based on sampling and not exhaustive, the best option is to use Clair3 with Illumina data for calling SNP and small indels up to 29 bp and use Sniffles2 with ONT data for calling bigger indels.

**Figure 1:**
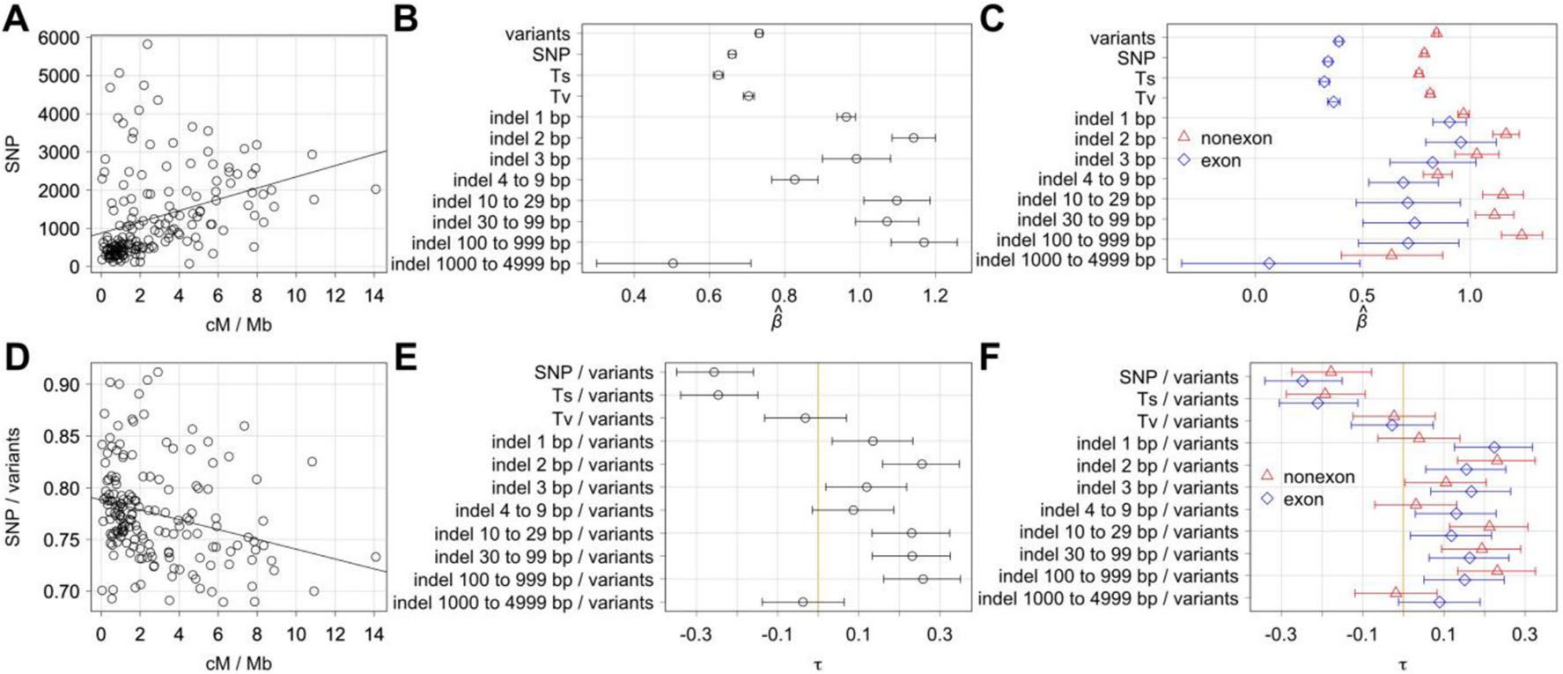
Recombination rate and abundance and proportion of variant subtypes. All analyses are of CB4856 HA with ONT and Illumina data with genomic intervals of ~ 0.6 Mb. (**A**) The number of SNP and the recombination rate of genomic intervals. (**B**) Regression coefficient $\widehat{\upbeta }$ and 95% confidence intervals between recombination rate and abundance of various variant subtypes. (**C**) Same as (**B**) separating variants that affect exons (blue) and variants that do not affect exons (red). (**D**) Proportion of SNP out of all variants and the recombination rate of genomic intervals. (**E**) Kendall tau and 95% confidence intervals between recombination rate and various proportions of variant subtypes. Kendall tau with 95% confidence interval spanning across tau of zero marked by orange line indicates absence of correlation. (**F**) Same as (**E**) separating variants that affect exons (blue) and variants that do not affect exons (red).

With the best data of CB4856 HA, there are more single nucleotide polymorphisms (SNP) in genomic intervals associated with higher recombination rate (Fig. 1A). We examined the correlation between recombination rate and the abundance of SNP and other variant subtypes by generalized linear modeling with Poisson distribution. There is a positive association between SNP abundance and local recombination rate (Fig. 1B). Association is weaker with transitions (Ts) and stronger with transversions (Tv). Notably, the association is considerably stronger with indels of 1 bp and indels of 2 bp, which are the most abundant indel subtypes. Among indels of other size changes, indels of 10–29 bp, indels of 30–99 bp, and indels of 100–999 bp show strengths of association comparable to indels of 2 bp whereas indels of other sizes show weaker associations. Similar patterns associations exist with SNP and indel subtype abundance using other variant callers and sequence data of other wild isolates (Supplementary Figs. 1 and 2).

Proportion of variant subtypes, such as SNP out of all variants and indels of 100–999 bp out of all variants, also could be used to assess differences in the correlation with recombination rate. With SNP out of all variants, there is a negative correlation with recombination rate (Fig. 1D) unlike positive correlations with many indel subtype proportions. The likes of Kendall rank coefficient^36^ could be used to assess these correlations. Notably, 95% confidence interval excluding a value of zero suggests a real positive or negative correlation. Many but not all variant subtype proportions show a strong correlation with recombination rate by this measure. With the best data of CB4856 HA, there is a strong negative correlation between recombination rate and SNP out of all variants and Ts out of all variants whereas there is a strong positive correlation between recombination rate and indels of 2 bp out of all variants, indels of 10–29 bp out of all variants, indels of 30–99 bp out of all variants, and indels of 100–999 bp out of all variants (Fig. 1E).

Covariates such as exon density, repetitive sequence density, GC content, gene expression, and methylation state and their relationship to the abundance and proportion of variant subtypes were examined as was done previously^10^. With Kendall rank coefficient examining the proportion of variant subtypes, the most striking correlations are with exon density although correlations are mostly absent with essential gene exon density (Supplementary Fig. 3). With other covariates, correlations are less striking overall with repetitive sequence density and most methylation states, and correlations are largely absent with GC content and gene expression level. Next, we further address exon and repetitive density.

Addressing the exon effect, we divided the variants into variants that affect or do not affect exons. With generalized linear modeling using Poisson distribution, association with recombination rate is stronger with variants that do not affect exon, and disparities between SNP and indel subtypes exist with both variants that affect and do not affect exon (Fig. 1C). Correlations between recombination rate and many variant subtype proportions also exist with both variants that affect and do not affect exon (Fig. 1F). Same conclusions could be made with analysis expanding exon boundaries by up to 100 bp (Supplementary Fig. 4). We also divided variants into variants that affect or do not affect repetitive DNA. Here, stronger correlations with recombination rate exist with many variant subtype proportions with variants that do not affect repetitive sequences (Supplementary Fig. 5). These observations are inconsistent with exon and repetitive sequence densities being the main driver of the correlations between recombination rate and many variant subtype proportions.

### A mutation model could explain the existing abundance and proportion of variant subtypes

Correlation between recombination rate and natural variant abundance was established long ago in CB4856 HA, and background selection was thought to be primarily responsible for this correlation^37, 38^. There is also reported evidence of selective sweep in *C. elegans* resulting in fewer haplotypes in regions of low recombination and thus fewer variants at population level^39^. However, variant subtype proportions are not affected by background selection and selective sweep; elaboration is provided in the last section of Results. A key feature of the mutation model is that it addresses disparities in both variant abundance and variant subtype proportions^10^.

The mutation model could be explained as follows. Mutation rate describes the frequency of new mutations. There are many ways of defining mutation rates, such as mutations per base pair per cell division or mutations per genome per generation. Within these definitions, there is a time component (e.g. one cell division or one generation) and a DNA component (e.g. one base pair or one genome). The mutation model uses the fact that DNA could be measured two different ways, specifically the physical distance (e.g. megabase or Mb) and the genetic distance (e.g. centimorgan or cM). The idea of a mutation rate that is dependent on the genetic distance is reasonable given that genetic distance depends on recombination frequency, and recombination is known to generate mutations.

The mutation model invokes two distinct groups of mutation mechanisms called Morgan and Sanger mechanisms. The Morgan mechanism is dependent on genetic distance, and the Sanger mechanism is dependent on physical distance. Each mechanism has its own mutation rate, which we call Morgan rate and Sanger rate. The Morgan rate is defined as mutations per genetic distance per time, and the Sanger rate is defined as mutations per physical distance per time. Time units based on biological cycles (e.g. generation) are more compatible with the mutation model, and one generation entails two meiosis and some larger numbers of mitosis. To determine the number of mutations, Morgan and Sanger mutation rates, genetic and physical distances of DNA, and time need to be accounted for. DNA variants are outcomes of mutations.

$$\begin{gathered} \# \;{\text{variants}}\;{\text{by}}\;{\text{Sanger}}\;{\text{mechanism}} = {\text{DNA}}\;{\text{interval}}\;{{\text{[Mb]}}}*{\text{time}}*{\text{Sanger}}\;{\text{rate}} \hfill \\ \# \;{\text{variants}}\;{\text{by}}\;{\text{Morgan}}\;{\text{mechanism}} = {\text{DNA}}\;{\text{interval}}\;{{\text{[cM]}}}*{\text{time}}*{\text{Morgan}}\;{\text{rate}} \hfill \\ \end{gathered}$$

Mutation rate could change depending on many factors, including the state of the genome and the physiological state of the organism. For example, fidelity of replication could be affected by changes in the DNA polymerase, and problematic conditions such as starvation hypothetically could lead cells to inactivate DNA repair machinery as a cost-saving measure. We are not especially concerned with the mutation rates at a specific time, but we are interested in the average mutation rate over a long period of time. Notably, we could define a coefficient that describes the ratio between the Morgan and Sanger rates. We call this R coefficient. With the R coefficient, Sanger rate could be redefined.

$$\begin{gathered} {\text{R}}\;{\text{coefficient}}\;{{\text{[cM}}\;{\text{Mb}}^{{ - {1}}}{\text{]}}} = {\text{Sanger}}\;{\text{rate}}/{\text{Morgan}}\;{\text{rate}} \hfill \\ {\text{Sanger}}\;{\text{rate}} = {\text{Morgan}}\;{\text{rate}}*{\text{R}}\;{\text{coefficient}} \hfill \\ \end{gathered}$$

With algebraic transformations (see Supplementary Text 1), the number of variants generated by Morgan and Sanger mechanisms could be described using the recombination rate (r) and physical distance (n) of DNA interval, R coefficient (R), Morgan rate (M), and time for divergence (d).

1 $$\begin{gathered} \# \;{\text{variants}}\;{\text{by}}\;{\text{Sanger}} = {\text{R}}\;{\text{coefficient}}\;{\text{(R)}}*{\text{DNA}}\;{\text{interval}}\;{\text{(n)}}*{\text{time}}\;{\text{(d)}}*{\text{Morgan}}\;{\text{rate}}\;{\text{(M)}} = {\text{R}}*{\text{n}}*{\text{d}}*{\text{M}} \hfill \\ \# \;{\text{variants}}\;{\text{by}}\;{\text{Morgan}} = {\text{DNA}}\;{\text{interval}}\;{\text{(r)}}*{\text{DNA}}\;{\text{interval}}\;{\text{(n)}}*{\text{time}}\;{\text{(d)}}*{\text{Morgan}}\;{\text{rate}}\;{\text{(M)}} = {\text{r}}*{\text{n}}*{\text{d}}*{\text{M}} \hfill \\ \# \;{\text{variants}}\;{\text{total}} = \left( {{\text{R}}*{\text{n}}*{\text{d}}*{\text{M}}} \right) + \left( {{\text{r}}*{\text{n}}*{\text{d}}*{\text{M}}} \right) = \left( {{\text{R}} + {\text{r}}} \right)*{\text{n}}*{\text{M}}*{\text{d}} \hfill \\ \end{gathered}$$

In addition, the model could be used to predict the number of a specific variant subtype. This is based on the idea that there is a specific probability of a new mutation being a specific mutation subtype. The probability is different for Morgan and Sanger mechanisms. For example, 1% of mutations by the Sanger mechanism could be indels of 5–10 bp whereas 2% of mutations by the Morgan mechanism could be indels of 5–10 bp. The same equation could be used with different variant subtypes, such as transitions, transversions, and indels of specific size.

2 $$\begin{gathered} \# \;{\text{indels}}\;{\text{by}}\;{\text{Morgan}} = {\text{probability}}\;{\text{of}}\;{\text{the}}\;{\text{variant}}\;{\text{being}}\;{\text{an}}\;{\text{indel}}\;\left( {{\text{F}}_{{\text{M}}} } \right)*{\text{r}}*{\text{n}}*{\text{d}}*{\text{M}} \hfill \\ \# \;{\text{indels}}\;{\text{by}}\;{\text{Sanger}} = {\text{probability}}\;{\text{of}}\;{\text{the}}\;{\text{variant}}\;{\text{being}}\;{\text{an}}\;{\text{indel}}\;\left( {{\text{F}}_{{\text{S}}} } \right)*{\text{R}}*{\text{n}}*{\text{d}}*{\text{M}} \hfill \\ \# \;{\text{indels}}\;{\text{total}} = \left( {{\text{F}}_{{\text{M}}} *{\text{r}}*{\text{n}}*{\text{d}}*{\text{M}}} \right) + \left( {{\text{F}}_{{\text{S}}} *{\text{R}}*{\text{n}}*{\text{d}}*{\text{M}}} \right) = \left( {{\text{F}}_{{\text{M}}} *{\text{r}} + {\text{F}}_{{\text{S}}} *{\text{R}}} \right)*{\text{n}}*{\text{M}}*{\text{d}} \hfill \\ \end{gathered}$$

The equations could be combined for the percentage of a variant subtype. For example, the equation for sum of number of indels by Morgan and Sanger mechanisms could be divided by the equation for sum of number of all variants by Morgan and Sanger mechanisms.

3 $$\begin{gathered} \% \;{\text{indels}} = \left( {\left( {{\text{F}}_{{\text{M}}} *{\text{r}} + {\text{F}}_{{\text{S}}} *{\text{R}}} \right)*{\text{n}}*{\text{M}}*{\text{d}}} \right)/\left( {\left( {{\text{R}} + {\text{r}}} \right)*{\text{n}}*{\text{M}}*{\text{d}}} \right) = \left( {{\text{F}}_{{\text{M}}} *{\text{r}} + {\text{F}}_{{\text{S}}} *{\text{R}}} \right)/\left( {{\text{R}} + {\text{r}}} \right) \end{gathered}$$

Here, larger F_M_ and greater probability of Morgan mechanism generating a specific variant subtype leads to stronger positive correlation between recombination rate and the abundance of a specific variant subtype.

Non-linear least squares (NLS) regression analysis could use the equations of mutation model to provide estimates of unknown values with known genomic interval values and variant data. As shown previously^10^, NLS analysis could provide estimates of R and ${\text{M}}*{\text{d}}$ product with Eq. (1) and estimates of R, F_M_, and F_S_ with Eq. (3). Larger R estimates suggest that recombination is a comparatively smaller source of natural variants, and larger ${\text{M}}*{\text{d}}$ estimates suggest that variants have been accumulating for a longer period of time. Here, we introduce two more methods of NLS analysis. First, Eq. (2) could be used to provide estimates of F_M_ and F_S_ after first estimating R with Eq. (1). Similarly, Eq. (3) also could be used to provide F_M_ and F_S_ after first estimating R with Eq. (1). These two-step approaches reliably provide estimates with small p values with a caveat that these p values are not equivalent to p values from one-step approach, which tries to identify three unknown values at once using Eq. (3) alone.

### Enhancing analysis with phylogenetic examination

Use of phylogenetic examination could enhance the analysis of variant abundance and variant subtype proportions. Phylogenetic examination could be used to classify DNA segments based on divergence from the reference DNA. More divergent DNA has a larger number of variants, which is useful with analysis of variant subtype proportions, especially rare variant subtypes. Phylogenetic examination could also identify distinct DNA families, which could be called haplotypes except that we want to highlight differences within each family. Each DNA family could be used as a single data point and thus increase sample size, and increased sample size leads to estimates with smaller p values in NLS analysis. This approach eliminates the effect of some intervals having more DNA families than others. Since DNA families could be eliminated by selective sweep but not background selection and mutation, effect of selective sweep could be separated. For all these reasons, we examined phylogenetic relationships among a set of 48 wild isolates using SNPs newly identified with Clair3.

**Figure 2:**
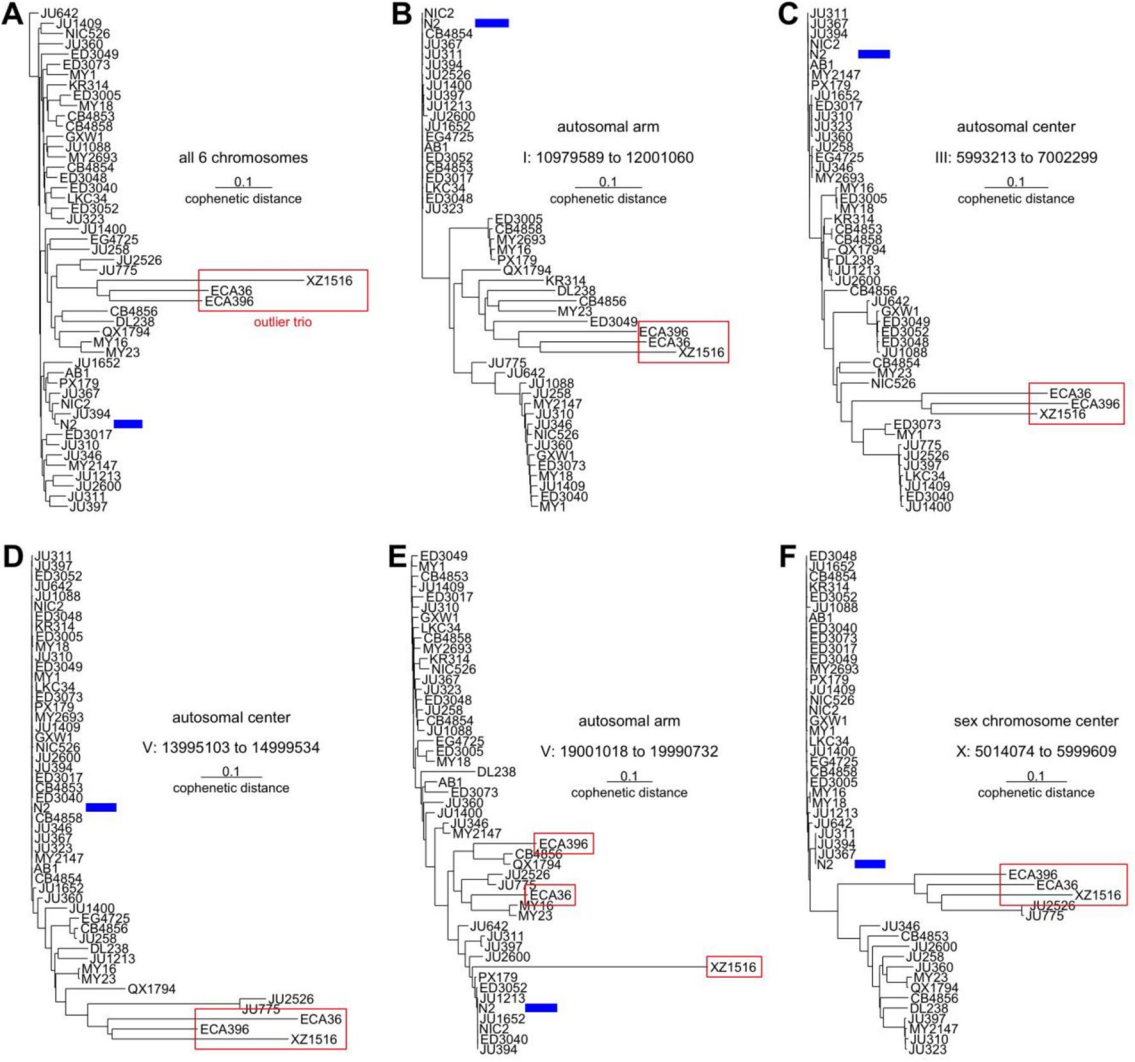
Phylogenetic relationships among DNA of N2 reference and 48 wild isolates. Neighbor-joining tree examining (**A**) all six chromosomes and ~ 1 Mb interval in (**B**) right arm of chromosome I, (**C**) middle of chromosome III, (**D**) middle of chromosome V, (**E**) right arm of chromosome V, and (**F**) middle of chromosome X. Outlier trio of ECA36, ECA396, and XZ1516 are marked by red rectangles. N2 reference is marked by blue bar.

**Figure 3:**
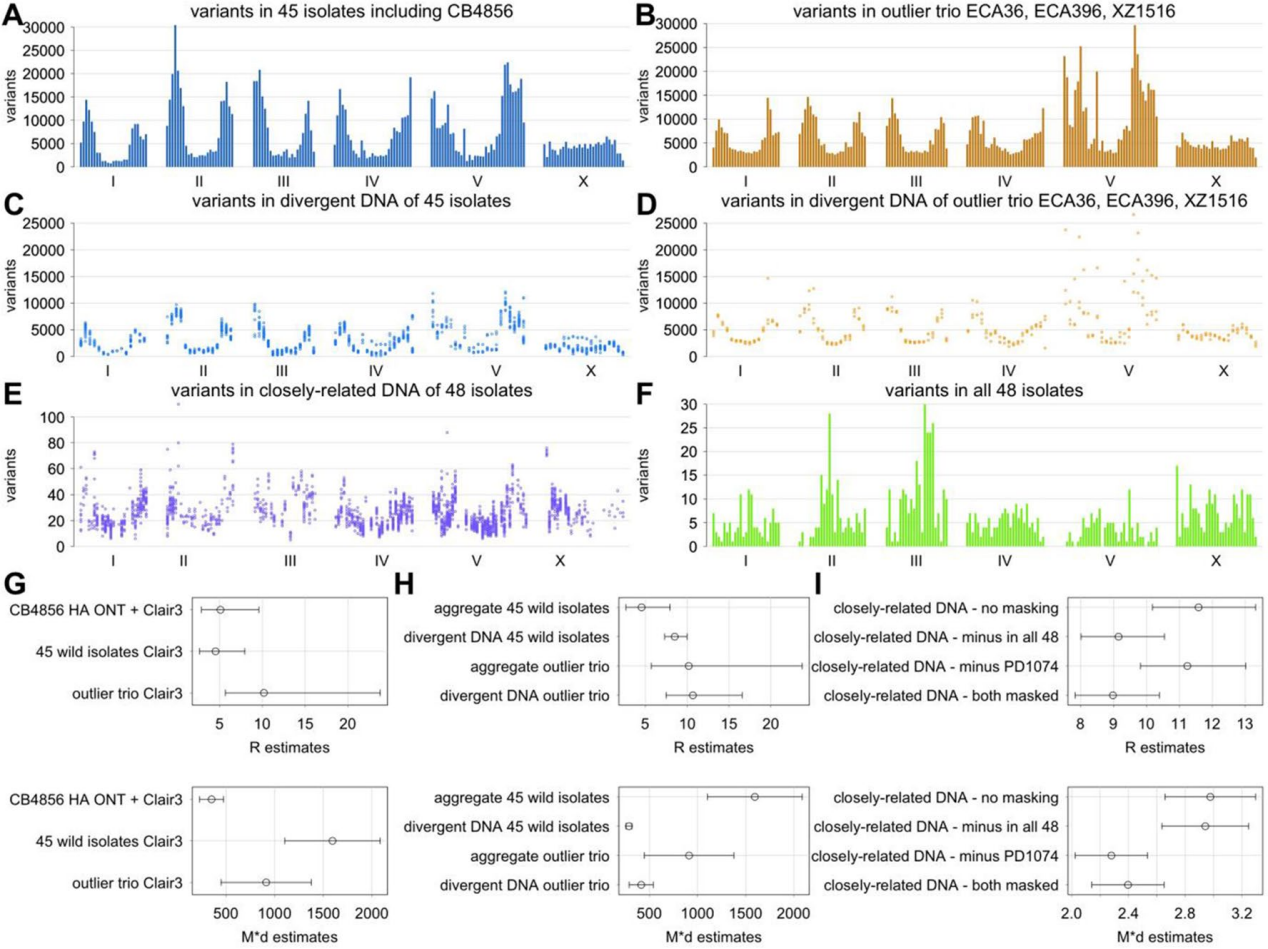
Analysis of variant abundance. (**A**-**F**) Number of variants in ~ 0.6 Mb intervals across six chromosomes are shown. (**A**-**B**) Total number of variants in aggregate (**A**) for 45 wild isolates without the outlier trio and (**B**) for the outlier trio of ECA36, ECA396, and XZ1516. (**C**-**D**) Mean number of variants in groups of divergent DNA (**C**) among the 45 isolates and (**D**) among the outlier trio. (**E**) Number of variants in closely-related DNA among all 48 isolates. (**F**) Number of variants that are present in all 48 isolates. (**G**-**I**) R (top) and ${\text{M}}*{\text{d}}$ (bottom) estimates and 95% confidence intervals are shown for different variant data sets. (**G**) Estimates using CB4856 HA with ONT data in addition to Illumina data, the 45 isolates in aggregate, and the outlier trio in aggregate. (**H**) Estimates using the 45 isolates and the outlier trio in aggregate and using divergent DNA within these groups. (**I**) Estimates using closely-related DNA in the 48 isolates without masking, with masking of variants that are present in all 48 isolates, with masking of variants that are present in PD1074, and with masking of both. Unless indicated otherwise, all analysis is of variants called by Clair3 using Illumina data.

Phylogenetic trees of wild isolates with many small DNA intervals look quite different as compared to a phylogenetic tree of whole genome (Fig. 2). Examining the length of branches from common ancestors to different wild isolates and their closest relatives, many branch lengths are similar in the tree of the whole genome (Fig. 2A). On the other hand with small intervals (Fig. 2B–F), there are distinct patterns of branch lengths, which could be used to place groups of DNA of wild isolates into distinct DNA families. Also with the whole genome as well as small intervals, a trio of outlier isolates ECA36, ECA396, and XZ1516, which have larger number of variants than the other 45 wild isolates (Fig. 3A,B), have considerably longer branches (Fig. 2A,C). The length differences are in general more pronounced in autosomal centers, which have low recombination rate, as compared to autosomal arms (Figs. 2B–E, 3C,D). Thus in addition to grouping DNA that are more divergent from the N2 reference from those that are closely-related to N2, there could be two distinct subsets of DNA within the set of divergent DNA. Enough concerns exist with the outlier trio with a huge number of variants, and thus some analyses were done with and without them.

Cophenetic distance was used to separate DNA that are closely-related to the N2 reference from DNA that are divergent. DNA segments with a small cophenetic distance in between are closely-related, and here we chose a minimum cophenetic distance of 0.01 as the upper limit for a close relationship. Depending on the choice of minimum cophenetic distance, classification could change among closely-related, divergent and a mixture of both. Separate analysis could be made with divergent DNA and with closely-related DNA. With divergent DNA, we first counted the number of variants within each distinct group, and then we calculated the arithmetic mean values to serve as data points. Thus for a DNA interval with five distinct groups of divergent DNA made up of ten isolates, the sample size is five rather than ten. With increased sample size, estimates with p values all below 0.05 using only Eq. (3) could be obtained, which has not been possible with simple aggregated variant data without phylogenetic examination.

### NLS regression analysis using the mutation model

With Eq. (1) of the mutation model, NLS regression analysis was done using several different variant data sets. With CB4856 HA, R estimate is 5 with 95% confidence interval from 3 to 10 (Fig. 3G). This R estimate is similar to R estimate of 5 (95% CI from 3 to 8) with 45 of 48 wild isolates in aggregate without the outlier trio of ECA36, ECA396, and XZ1516. With the outlier trio, R estimate is 10 (95% C. I. from 6 to 24), which likely reflects greater magnitude of increases in the number of variants in autosomal centers with low recombination rate as compared to autosomal arms (Fig. 3A–D). Relative importance of Morgan and Sanger mechanisms could be assessed using the R value along with genetic and physical distances spanning the genome (Supplementary Text 1). R of 5 suggests that 36% (95% C. I. from 26 to 48%) of all variants in natural populations were generated by the Morgan mechanism, and thus R of 5 would mean that meiosis and recombination have a substantial role in mutation generation in natural populations.

Effect of phylogenetic examination on NLS regression analysis is examined next. With divergent DNA in 45 of 48 wild isolates in aggregate without the outlier trio of ECA36, ECA396, and XZ1516, R estimate rises to 8 (95% C. I. from 7 to 10) (Fig. 3H). This effect is likely caused largely by the presence of larger numbers of distinct DNA families in regions of high recombination. Similar rises in R estimates are seen also with all 48 wild isolates as well as with many smaller subsets of wild isolates (Supplementary Fig. 6) while this rise is fairly small with only the outlier trio of ECA36, ECA396, and XZ1516, which are quite different from each other for much of the genome.

DNA that are closely-related to N2 reference also have a skewed variant abundance with more variants in autosomal arms (Fig. 3E). With NLS regression analysis using raw data, the R estimate is 12 (Fig. 3I), which is somewhat larger than the R estimate with divergent DNA of 45 wild isolates. We think that masking is needed with closely-related DNA to account for N2-specific variants and problems with the N2 reference genome sequence (see Discussion). Masking is a significant factor because of the relatively small number of variants in the closely-related DNA data set. Two methods were used. First, we removed variants that are present in all 48 isolates and are thus considered to be N2-specific, and these variants are enriched in autosomal centers (Fig. 3F). Second, variants that are present in an N2-derivative PD1074 were removed. The R estimate with masking is 9 (Fig. 3I), which is similar to the R estimate with the divergent DNA of 45 wild isolates (Fig. 3H).

**Figure 4:**
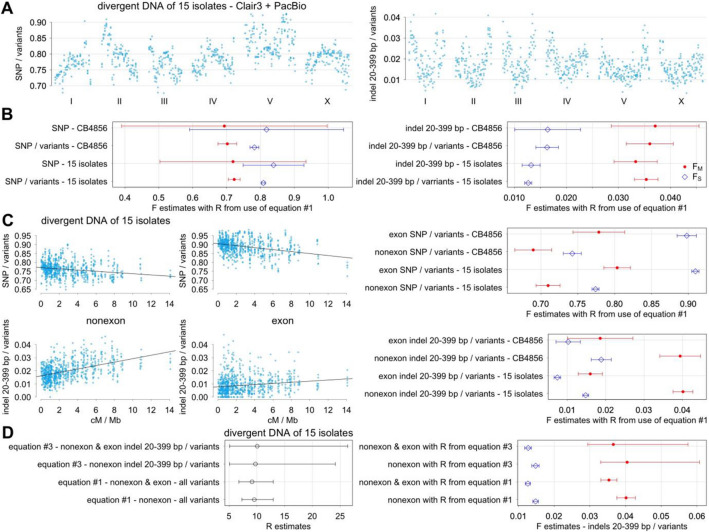
Analysis of variant subtype proportions. Variant subtype abundance with SNP and indels of 20–399 bp and variant subtype proportions of SNP out of variants and indels of 20–399 bp out of variants are examined. Variant data are of CB4856 HA with ONT and Illumina data and divergent DNA of 15 wild isolates with PacBio and Illumina data. (**A**) Mean proportions of SNP out of variants (left) and indels of 20–399 bp out of variants (right) across ~ 0.6 Mb intervals in six chromosomes. (**B**) F estimates with 95% confidence intervals. (**C**) Same proportions as (**A**) separated by variants that affect or do not affect exons are plotted against the recombination rate of genomic intervals on the left. F_M_ and F_S_ estimates with 95% confidence intervals separating variants that affect or do not affect exons on the right. (**D**) R, F_M_, and F_S_ estimates using Eq. (3) examining indels of 20–399 bp out of variants are compared against R estimates using Eq. (1) on the left. F_M_ and F_S_ estimates using Eq. (3) alone are compared against the same using Eq. (1) for R prior to calculating F_M_ and F_S_ using Eqs. (2) and (3).

F_M_ and F_S_ estimates could be obtained with Eqs. (2) and (3) of the mutation model by NLS regression analysis after first estimating R value using Eq. (1). With all variants as the denominator in Eq. (3), F_M_ and F_S_ values corresponding to a variant subtype proportion are identical to F_M_ and F_S_ values corresponding to the same variant subtype abundance in Eq. (2). Thus, analysis with proportions of SNP out of variants in different genomic intervals (Fig. 4A) is equivalent to SNP abundance analysis. With both analyses, F_M_ and F_S_ estimates are 0.7 and 0.8, respectively, with CB4856 HA data with R estimate of ~ 5 (Fig. 4B). With divergent DNA of 15 wild isolates with PacBio data and R estimate of ~ 9, F_M_ and F_S_ estimates are also 0.7 and 0.8, respectively. Thus even with different R values, F_M_ and F_S_ estimates from different data sets are similar. Similarly with indels of 20–399 bp and indels of 20–399 bp out of variants, F_M_ and F_S_ estimates are from 0.03 to 0.04 and from 0.01 to 0.02, respectively. Comparisons were made with many variant subtypes, and estimates are similar in all cases (Supplementary Fig. 7).

Proportions of various variant subtypes generated by the Morgan mechanism could be assessed using the values of R, F_M_, and F_S_ along with genetic and physical distances of the genome (Supplementary Text 1). With the estimates of F_M_ of 0.7 and F_S_ of 0.8 with SNP, Morgan mechanism is expected to be responsible for 32–33% of SNP (95% CI from 17 to 48%) with R of 5 using the CB4856 HA data and 21–22% of SNP (95% CI from 14 to 28%) with R of 9 using the 15 wild isolate data. With F_M_ and F_S_ estimates of indels of 20–399 bp, contribution from Morgan mechanism is expected to be 55% (95% CI from 41 to 71%) with CB4856 HA and 44–46% (95% CI from 37 to 50%) with 15 isolates. In general, the mutation model suggests that recombination has a smaller role with SNP and a larger role with indels.

Effect of exons on F_M_ and F_S_ estimates is examined next. SNP is more common in exons whereas indels are less common (Fig. 4C). Consequently with SNP, F_M_ and F_S_ estimates are considerably larger with variants that affect exons whereas F_M_ and F_S_ estimates are somewhat smaller with variants that do not affect exons. With indels of 20–399 bp instead, F_M_ and F_S_ estimates are considerably smaller with variants that affect exons whereas F_M_ and F_S_ estimates are somewhat larger with variants that do not affect exons. Proportions of indels are larger outside exons, and F_M_ and F_S_ estimates are usually larger with variants that do not affect exons (Supplementary Fig. 8). In general, F_M_ and F_S_ estimates with variants that affect exons are quite different as compared to those with all variants and with variants that do not affect exons, and this is probably because variants that affect exons are sparsely distributed and are a small minority of all natural variants.

Simultaneous estimation of R, F_M_, and F_S_ is possible with Eq. (3) using NLS regression analysis. Bigger sample size, which could be obtained by phylogenetic examination, is necessary for high-quality estimates with p-values below 0.05. With an example of indels of 20–399 bp out of all variants, R, F_M_, and F_S_ estimates are all quite similar regardless whether R is estimated using Eq. (3) or Eq. (1) (Fig. 4D). On the other hand with a large difference in R estimates from Eq. (3) and from Eq. (1), there tends to be a greater divergence in F_M_ and F_S_ estimates. Ideally, R estimates from Eqs. (3) and (1) should be similar, but there are considerable differences depending on the size of genomic intervals as addressed next.

Effect of genomic interval size on variant abundance analysis was examined with interval sizes from 0.2 Mb to 2 Mb and from 2 to 5 cM (Supplementary Fig. 9). With Eq. (1) of the mutation model, R estimates are smaller with larger intervals with all variant data sets. With some data such as divergent DNA of 45 wild isolates, there is no overlap between 95% confidence intervals of R estimate using interval sizes of 0.2 Mb and 1 Mb. Notably, interval size also has a strong effect on NLS regression analysis using equations of selective sweep and background selection. We note that genetic distances are based on WormBase map positions, which were determined with classic mapping only; perhaps incorporating genomics approach to study recombination^40, 41^ to refine genetic positions could be useful. With Eq. (3) of the mutation model examining variant subtype proportions, there is no trend of smaller R estimates with larger intervals (Supplementary Fig. 10). R estimates using Eqs. (1) and (3) should be similar, and we chose 0.6 Mb as the smallest interval size that meet this standard.

### Analysis combining the mutation model with linked selection models

The mutation model could explain the correlation between recombination rate and variant abundance, which has been considered primarily as the consequence of background selection in *C. elegans*^37, 38^ with selective sweep also playing a role^39^. With species with high inbreeding rate, we could use the following versions of mathematical equations of selective sweep and background selection as was shown^37^, which are adapted from other earlier works^7, 42–45^.

$$\begin{gathered} {\text{selective sweep}}: \pi = \pi_{{\text{o}}} *{\text{r}}*\left( {{1} - {\text{F}}} \right)/\left( {\left( {{1} + {\text{F}}} \right)*\left( {{\text{r}}*\left( {{1} - {\text{F}}} \right) + \beta } \right)} \right) \hfill \\ {\text{background selection}}: \pi = \pi_{{\text{o}}} *{\text{exp}}\left( { - {\text{U}}/\left( {{\text{s}}_{{\text{d}}} + {\text{r}}*\left( {{1} - {\text{F}}} \right)} \right)/\left( {{1} + {\text{F}}} \right)} \right) \hfill \\ \end{gathered}$$

These describe how selective sweep and background selection change π_ο_, which is expected variant abundance without linked selection models, into π, observed variant abundance. Factors relevant to both are (r) recombination rate and (F) inbreeding coefficient. With selective sweep, β is equal to $2*\text{N}_{\text e}*\text{s}_{\text a}*\text{v}_{\text o}*\text{k}$ with following definitions: (N_e_) effective population size, (s_a_) average selection coefficient for beneficial mutations, (v_o_) expected number of beneficial mutations, and (k) an integral with value approximately 0.075^46^ and also referred to as − I_M_^42^. With background selection, U is deleterious mutation rate, and s_d_ is the average selection coefficient for deleterious mutations, and exp is exponential function. These equations describe the likelihood of nearby neutral mutation removal depending on the recombination rate near the selected loci and other factors. Changes in the values of relevant factors such as average selection coefficient, inbreeding coefficient, and recombination rate affect neutral variant abundance. Neutral indel and neutral SNP of the same distance from the selected loci are affected equally. Thus, selective sweep and background selection do not explain the correlations between recombination rate and variant subtype proportions.

**Figure 5:**
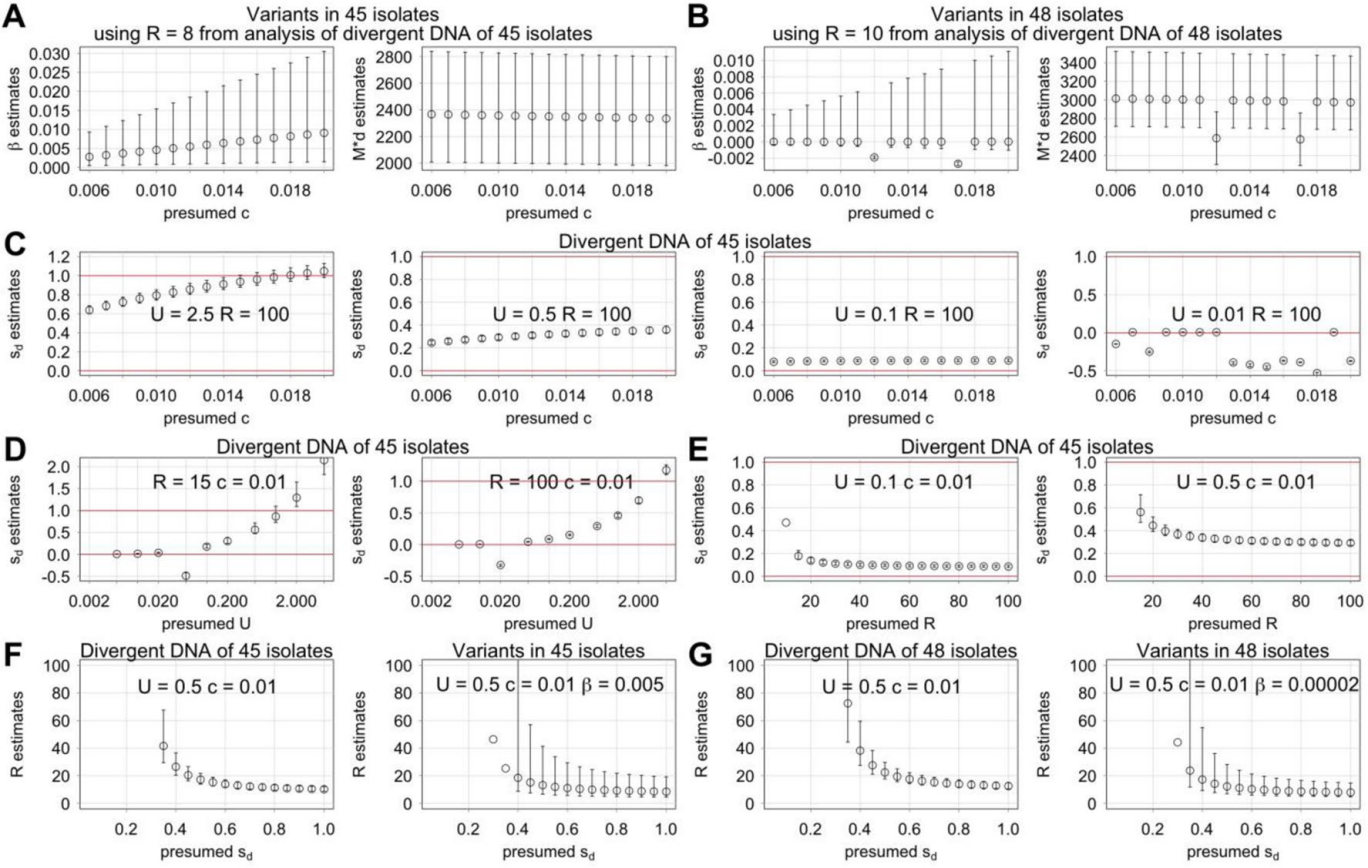
Analysis combining the mutation model with selective sweep and background selection. (**A**-**B**) Estimates of β (left) and ${\text{M}}*{\text{d}}$ (right) with 95% confidence intervals using variant data sets (**A**) of 45 wild isolates and (**B**) of 48 wild isolates including the outlier trio. (**C**) Estimates of s_d_ using a series of c values along with R of 100 and U of 2.5, 0.5, 0.1, and 0.01. Red lines indicate the upper and lower bounds of reasonable s_d_ values. (**D**) Estimates of s_d_ using U values 0.002, 0.005, 0.01, 0.02, 0.05, 0.1, 0.2, 0.5, 1, 2, and 5 along with c of 0.01 and R of 15 (left) and 100 (right). Outlier estimates far below 0 are not shown. (**E**) Estimates of s_d_ using a series of R values along with c of 0.01 and U of 0.1 (left) and 0.5 (right). Estimates far above 1 are not shown. (**F**-**G**) R estimates are compared between those obtained using the mutation model combined with background selection and using variant data with phylogenetic examination (left) and those obtained using the mutation model combined with both selective sweep and background selection and using variant data without phylogenetic examination (right). β values are from (**A**-**B**), and R estimates far above 100 are not shown. (**F**) Analysis using the 45 wild isolates. (**G**) Analysis using the 48 wild isolates.

Equations of selective sweep and mutation model could be combined by substituting π_ο_ with Eq. (1) of the mutation model. Because only selective sweep equation is affected by effective population size, we think that variant data with and without phylogenetic examination could be used to separate the effects of selective sweep and mutation. For example, variant data of 45 isolates without phylogenetic examination could be analyzed using R estimate with phylogenetic examination to obtain β and ${\text{M}}*{\text{d}}$ estimates; R estimates with and without phylogenetic examination are 8.5 and 4.5, respectively; this difference probably exists because phylogenetic examination masks the effect of greater number of haplotypes in regions of high recombination. Reported c value is in a range from 1% to 1.7%^47–49^ in *C. elegans* with an outlier value of 22%^50^. Presuming c of 0.01 in NLS analysis, β estimate is 0.005 (95% CI from 0.0007 to 0.02), and larger c leads to larger β estimates (Fig. 5A). With variant data of different numbers of wild isolates, β estimate is as high as 0.006 and as low as − 0.00008 with presumed c of 0.01, and larger c does not always lead to larger β estimates (Fig. 5B, Supplementary Fig. 11). Negative β value does not make sense, and most β estimates of a negative values have 95% confidence intervals that are mostly of positive β value. All things considered, correct β value likely is a small number well below 0.1 and above 0.

With background selection, neutral mutation is affected by background selection only if there is an existing linked deleterious mutation already when the neutral mutation arises. Thus, the strength of background selection depends on the preponderance of deleterious mutations that are present in a typical individual. Deleterious mutation rate U for *C. elegans* has been proposed to be in a range from 0.003 to 0.48 with 0.48 being the most recent estimate and the only estimate over 0.1 in primary literature^51–56^, and a review paper suggested an even higher U value of 2.5^57^. Equations of the mutation model and background selection could be combined^10^ as was done with selective sweep. Selection coefficient s_d_ could be estimated by NLS analysis with known or presumed values of U, c, and either R or ${\text{M}}*{\text{d}}$, and R and ${\text{M}}*{\text{d}}$ estimates could be made with presumed s_d_ values along with U, and c. Identifying a reasonable set of values for these variables could be useful rather than examining all possible values, and this could be aided by the limits of various values as described next.

**Figure 6:**
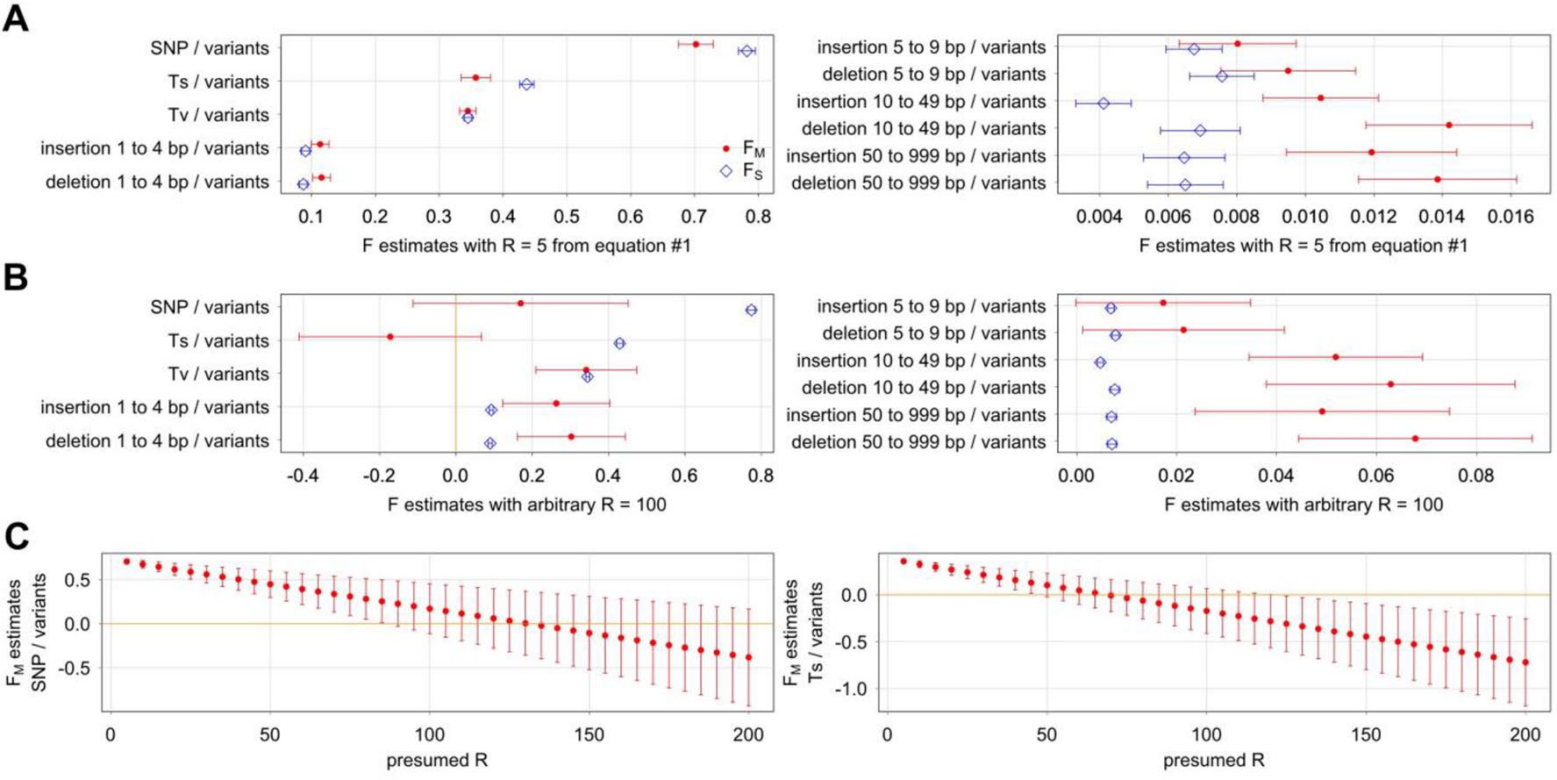
Upper limit of R value that could explain the correlation between recombination rate and all variant subtype proportions. F_M_ and F_S_ estimates and 95% confidence intervals are shown for variant subtype proportions including SNP out of variants, Ts out of variants, Tv out of variants, and insertions and deletions of various size ranges out of variants. Variant data set of CB4856 HA with ONT and Illumina data and genomic intervals of ~ 0.6 Mb are used. (**A**) Estimates with R value calculated using Eq. (1). (**B**) Estimates with arbitrary R value of 100. Orange line at F value of 0 indicates lower limit of realistic F_M_ and F_S_ values. (**C**) Estimates of F_M_ values of SNP out of variants (left) and Ts out of variants (right) with a series of arbitrary R values.

To identify the limits of R value that could explain the correlations between recombination rate and all variant subtype proportions, the effect of changing the R value was explored. F_M_ and F_S_ values like c and s_d_ are by definition between 0 and 1. Thus, R values that lead to unreasonable F_M_ and F_S_ values could be considered wrong, which could be used to identify a range of reasonable R values. With R of 5 using Eq. (1) of the mutation model and thus assuming no background selection effect, F_M_ and F_S_ estimates are 0.7 and 0.8, respectively, with SNP out of all variants using CB4856 HA data (Fig. 6A). Here, R of 5 could be considered the lower limit. With larger R values, F_M_ estimates for SNP are smaller. Similar trend exists also with F_M_ estimates of Ts out of all variants. With an R value of 100, F_M_ estimate of Ts out of all variants is smaller than zero (Fig. 6B), which is outside the reasonable range. With even larger R values, zero could be outside the range of 95% confidence interval of F_M_ estimates with Ts and SNP (Fig. 6C). Similar conclusions could be made with variant data of 15 isolates with PacBio data (Supplementary Fig. 12). We picked 100 as a convenient compromise for the upper limit of R for assessing variables of background selection next.

NLS analysis was done combining the mutation model and background selection with presumptions using reported U and c values with reasonable values of R and s_d_. With R at the upper limit of 100, there are wide ranges of s_d_ estimates (Fig. 5C). With large U values such as 2.5 and larger c values, s_d_ estimates could be over 1, which is nonsensical, and small U values such as 0.01 could lead to unreasonable s_d_ estimates under 0. We think that trends of unreasonable estimates indicate that presumed values are incompatible with the combined model. With a smaller R value of 15, large U values such as 2.5 lead to nonsensical s_d_ estimates and are incompatible (Fig. 5D). With moderate U values, reasonable s_d_ estimates exist with a wide range of R values (Fig. 5E); exceptions include all R values smaller than the R estimates with the mutation model alone and some R values slightly larger than these R estimates. To summarize, wide ranges of U, c, and R values are compatible with NLS analysis combining the mutation model and background selection.

Finally, the mutation model could be combined with both background selection and selective sweep by nesting, which increases the number of variables and presumptions for NLS analysis. One option is to use the β estimate obtained using the model combining mutation and selective sweep for estimating other unknown values in analysis combining all three models. For example, a set of R estimates could be obtained using variant data without phylogenetic examination with a set of presumed U, c, and β values along with a series of s_d_ values. These R estimates could be compared with R estimates obtained with the same set of U, c, and s_d_ values and using variant data with phylogenetic examination. With these parallel analyses, R estimates are similar (Fig. 5F,G). These similarities suggest that our method of combining mutation model with background selection and selective sweep is sound.

## Discussion

Natural selection could also affect variant subtype proportions, and this effect is evident with exons. Among variants identified with Clair3 in the 48 wild isolates, SNP comprises 91% of variants that affect exons whereas SNP comprises 77% of variants that do not affect exons (Supplementary Table 1). Indels are less common in exons, presumably because indel is more likely to disrupt protein coding than SNP. Generally, the number of indels is smaller when the indel size is bigger. However among indels that affect exons, indels of 6 bp are 1.5 times more prevalent than indels of 5 bp. Also, indels of 3 bp number 65% that of indels of 2 bp among variants that affect exons as opposed to 38% among variants that do not affect exons. These differences likely stem from the fact that indels of 3 bp and 6 bp do not lead to frameshift. Less clear is whether natural selection could explain the correlation between recombination rate and variant subtype proportions as addressed next.

Mechanisms underlying the connection between recombination rate and variant subtype proportions are difficult to fathom with a selection model. To bridge a connection, other covariates also correlated with recombination rate could be considered. Exons occupy over 40% of DNA in some intervals, and a negative correlation between recombination rate and gene density is well-known^58^. Outside exons, natural selection likely has a strong effect on DNA elements controlling gene expression. However, masking by removal of variants that affect exon and repetitive sequences actually leads to stronger correlations between recombination rate and many variant subtype proportions (Fig. 1 and Supplementary Figs. 4 and 5). Furthermore, correlations do not change substantially by expanding masked DNA to include the likes of splice acceptors and donors and beyond. Thus, there is little rationale for making a connection using exons and repetitive sequences. While the mutation model may yet turn out not to be the primary explanation for the correlation between recombination rate and variant subtype proportions, we are unable to conceive a compelling alternative.

Reliability of estimates from NLS regression analysis using the mutation model is addressed next. With divergent DNA of a common set of wild isolates, R estimates are similar between the new variant data and the old MMP data; R estimate is 8 both with 45 wild isolates (Fig. 3H) and with the MMP data. While changes to the set of wild isolates (e.g. outlier trio ECA36, ECA396, XZ1516) lead to larger changes, 95% confidence intervals still overlap (Supplementary Fig. 6). R estimates are quite similar in analysis with and without accounting for exons while F_M_ and F_S_ estimates change to a greater degree. Choice of genomic interval size and orientation could eliminate the overlaps in 95% confidence intervals, however, the same is true with linkage selection models (Supplementary Fig. 9). Reliability of NLS regression analysis is equivalent with mutation and linkage selection models.

Censoring N2-specific variants has a large effect on the analysis of closely-related DNA. Small sample size is likely responsible, and we note that the R estimate is considerably higher using the MMP data instead. Prior to PD1074 sequencing, we relied solely on identifying variants that are present in all wild isolates. For example with the MMP data of 40 wild isolates, there are 677 variants that are present in all 40. With the new variant data of 48 wild isolates and 1,958,669 unique Clair3 variants, there is a common set of 1006 variants in all 48. Increase of variants common to all likely stems from a lower false negative variant calling error with Clair3. With PD1074, 607 of 3492 PD1074 variants that are present also in all 48 wild isolates have strange characteristics described next.

Unlike natural populations, the 607 PD1074 variant subset is not enriched in autosomal ends associated with high recombination and has a low SNP proportion (31%, n = 191 of 607, Supplementary Table 1 and Fig. 13). Low SNP proportion (37%, n = 58 of 161) exists also with PD1074 variants that are present in 47 of 48 wild isolates; false negatives could be responsible for the absence in single isolates. History of the N2 genome sequence is useful in discussion of these variants. Genome sequencing of N2 reference strain was performed primarily using cosmid libraries^59^, which were made in the 1980s using continuously cultured N2 nematodes (personal communication, John Sulston and Robert Waterston) along with yeast artificial chromosomes, which were made from an endonuclease mutant strain CB1392 *nuc-1(e1392)* derived from N2 by EMS mutagenesis (personal communication, RW). Thus, the methods used in N2 reference genome sequencing including mutations induced by EMS in CB1392 could be responsible for the peculiarities of these variant subsets.

SNP proportion and variant abundance similar to natural populations exist with 1372 PD1074 variants that are absent in all 48 wild isolates; these could be PD1074 private variants. Here, SNP is common (83%, n = 1137 of 1372, Supplementary Fig. 13), and variants are enriched in autosomal ends associated with high recombination. NLS regression analysis was done with only the presumptive 1372 PD1074 private variants. With these, the R estimate is 4, which is similar to R of 5 with CB4856 HA and R of 4 with divergent DNA without subsets of outlier isolates ECA36, ECA396, XZ1516, JU2526, and JU775, which have atypically larger number of variants in autosomal centers. Such similar R estimates are consistent with similar mutation rates in natural populations and PD1074.

Mutation accumulation (MA) strains need to be addressed. Weak positive correlation between recombination rate and variant abundance was reported in one MA study^60^, which examined ~ 3500 mutations from over 100 populations, which underwent a total of ~ 24,950 generations or 249.5 years assuming 100 generations per year. R estimates with this MA data are much higher as compared to natural populations and PD1074. There is also another MA data^61^ with ~ 3050 mutations from 86 MA lines, which underwent 409 generations each, meaning a total of ~ 35,000 generations. Notably, the number of generations involved in divergence between N2 and PD1074 is likely to be considerably smaller. According to one study, up to a thousand neutral mutations could have accumulated over 300–2000 generations from the initial isolation of N2 in 1951 to cryogenic preservations starting in 1969^62^. Meanwhile, there are ~ 1372 PD1074 private variants and a less clear number of N2 private variants. Resolving the differences with MA studies could entail further analysis including use of the same set of reliable variant callers and further assessment of variant calling quality.

Variant calling error remains with the current best methods, and both false positives and false negatives need to be considered. The effect of variant calling error is visible in phylogenetic trees with branches of different lengths of cophenetic distances within distinct DNA families (Fig. 2). These branch lengths are longer within DNA families that are divergent from N2 reference, and the difference is greater in intervals with many variants. This trend is more pronounced with the MMP data. Variant calling is more problematic with bigger indels using Illumina data and Clair3 and with small indels using ONT data and Sniffles2. With indels of 20–29 bp, both methods led to questionable variant calls, and we chose Illumina and Clair3 combination because of better precision. Future improvements could change some conclusions, especially with data of small sample size.

We confirmed the existence of the correlation between variant abundance and variant subtype proportions in *C. elegans* natural populations with the improved variant data. The mutation model explains this correlation, and future analysis may identify other possible explanations. Unlike an idea of mutation rate simply being elevated in regions of high recombination^63^, a more nuanced analysis is possible with a model of mutations controlled by genetic distance involving meiosis and mutations controlled by physical distance during other parts of cell cycle and mitosis. A new comprehensive approach to the analysis of natural variant data is outlined with our use of phylogenetic examination and our approach in combining the mutation model with linkage selection models.

## Methods and materials

Whole-genome sequence data are from BioProjects PRJNA523481, PRJNA549503, PRJNA764925 with CB4856 Illumina, ONT, and PacBio HiFi data, BioProject PRJNA692613 with 14 wild isolates with first-generation PacBio data, BioProject PRJNA523481 with Illumina data of select 47 wild isolates used in the main analysis, and BioProject PRJNA430756 with PD1074 Illumina data.

Alignments to WS245 *C. elegans* reference genome fasta file with appropriate index file were made using Minimap2 (v.2.17) with preset -x sr for Illumina fastq files and with preset -map-ont for ONT fastq files and using pbmm2 (v.1.10.0) for PacBio fastq files. Using SAMtools (v1.15.1), aligned SAM files were converted to BAM files, which were then sorted and indexed. Variant calls with Clair3 (clair3-arm64) were made using --platform="ilmn" and ilmn model. Mac M1 installation of Clair3 was done as instructed at https://github.com/HKU-BAL/Clair3/issues/149. Variant calls with BCFtools (v1.15.1) mpileup was done using --config Illumina while listing all relevant BAM files for simultaneous variant calling for each wild isolate, and vcf files were obtained using BCFtools call using the option --multiallelic-caller. Variant calls with GATK (v3.8) haplotypecaller was made using all relevant BAM files for simultaneous calling following the use of GATK AddOrReplaceReadGroups --RGLP lib1 --RGPL ILLUMINA --RGPU unit1 --RGSM sra.ids.samples to make BAM files usable with haplotypecaller. Variant calls with DeepVariant (v1.4.0) was made using --model_type WGS with merged BAM files; merging of BAM files were done for each wild isolate using SAMtools prior to variant calling with DeepVariant, Dysgu, SVIM, and Sniffles2. Variant calls with Dysgu (v1.3.14) were made using --mode nanopore and --mode pacbio with ONT BAM files and merged PacBio BAM files, respectively. Variant calls with SVIM was made with the option --min_sv_size 10. Variant calls with Sniffles2 (v.2.0.7) was made with the option --minsvlen 20, which led to identification of identification of indels of 20–29 bp as well as identification larger number of indels of larger than 30 bp as compared to --minslven 30 with ONT and PacBio BAM files.

Further screening of called variants was done using the variant output files as described next. With Clair3, we screened for GT value of 1/1 and PASS value for FILTER. With BCFtools mpileup, four DP4 values indicating the numbers of REF and ALT calls were first used to screen for SNP with at least 95% ALT, indels of 1–9 bp with at least 75% ALT, and indels of 10 + bp with at least 60% ALT. Then, low-quality variants with AC value with half or less maximum value were removed (e.g. AC = 6 kept with maximum AC = 10). With GATK haplotypecaller, we screened for variants with AC value of greater than half the maximum AC value and of even number for AC, and then variants with multiple ALT alleles were removed. With DeepVariant, we screened for variants with GT value of 1/1, and then variants with multiple ALT alleles were removed. With Sniffles2, we screened for variants with GT value of 1/1 and of variant subtypes INS and DEL. With Dysgu, we screened for variants with GT value of 1/1 and variant subtypes INS and DEL first. Next, we examined the supporting evidence value SU and average coverage value COV, and variants with SU/COV value less than 0.75 were removed. With SVIM, we screened for variants with GT values of 1/1. Quality of variant calling was evaluated by visual examination of a subset of samples using Integrated Genome Viewer (v2.12.2).

For the main analysis of the variant data, custom scripts were written and executed using R^64^, R studio^65^, and macOS terminal. Some procedures previously used^10^ for processing of variants were omitted. These include joining two or more variants that affect adjacent base pairs or share the same start or end position in a common wild isolate and removing variants that overlap and variants inside other variants, which removed variants in regions now referred as hyperdivergent^19^ from the analysis.

Updated *C. elegans* variant data and genomic features were extracted from gff3 file of WormBase^66^ WS287 PRJNA13758. Terms used for screening with AWK include gene, exon, CDS, RNASeq_reads, mRNA, and repeat with secondary terms WormBase and RepeatMasker for additional screening. To obtain a list of essential genes with WormMine version WS287 (http://intermine.wormbase.org/tools/wormmine/begin.do), we used the queries lethal, sterile, larval lethal, larval arrest, embryonic lethal, and zygotic lethal as the mutant phenotypes. WIG files for chromatin state by modENCODE project^67^ are used unchanged from our previous work^10^ except to account for different genomic interval boundaries.

With the size of genomic intervals, we targeted sizes in a range from ~ 0.2 Mb to ~ 2 Mb in 0.1 Mb increments and from ~ 2 cM to ~ 5 cM in 0.2 cM increments. To define the ends of genomic intervals, we used genes with interpolated genetic locations. There are over 20,000 genes with an interpolated genetic location, and using these genes allows a more precise definition of genetic distance, physical distance, and recombination rate of all intervals. For example, with the first 1 Mb interval from left to right of chromosomes, we identified genes that are nearest the nucleotide positions 1 and 1,000,000, and we used the left end of the gene nearest position 1 as the left end of the first interval and the left end of the gene nearest position 1,000,000 as the left end of the second interval, respectively. We also used intervals starting from the right end and ending with the left end of chromosomes. Because the last interval for each chromosome could be considerably smaller than the targeted interval size, we combined the last interval with the penultimate interval if the size of the last interval is smaller than half of the targeted size. Also, 999 intervals of 100 kb from the left end of each chromosome and 6 intervals of < 100 kb at the right ends of chromosomes were used for phylogenetic examination.

For correlation tests of variant abundance, generalized linear modeling with the Poisson distribution was used. To do this with base R, glm was used with the family poisson. For correlation tests of variant subtype proportion, we used Kendall rank correlation test^36^ using cor.test in base R with method kendall, and 95% confidence interval was calculated using a separate script tau.ci (https://rpubs.com/seriousstats/616206).

To perform NLS regression analysis^68, 69^, we used the R package minpack.lm^70^ using the Levenberg–Marquardt algorithm^71, 72^. Also, we tried using nls of base R with the port algorithm based on a FORTRAN program nl2sol^73, 74^, which could place arbitrary upper and lower limits on the estimates of R, F_M_, and F_S_. By definition, the value of R must be greater than zero, and the values of F_M_ and F_S_ must be between 0 and 1. While use of the port algorithm avoids estimates outside these ranges, the replacement estimates are equal to the limits (e.g. the replacement F_M_ estimate is 0 or 1), and the associated p values are > 0.99. Aside from the changes caused by the limits, the choice of algorithm has tiny or no effect on the value of the estimates obtained. The Levenberg–Marquardt algorithm is less finicky and is generally better at obtaining estimates of R, F_M_, and F_S_. The Levenberg–Marquardt algorithm is used with all analysis shown in this manuscript.

For NLS analysis using only the variants that affect or do not affect exons, we changed some parameters. Previously^10^, we subtracted the length of exons in determining the physical length of DNA intervals in analysis using variants that do not affect exons, and we now consider this method inappropriate. Instead, we presume that variant abundance would have been the same in the masked DNA as in non-masked DNA if all DNA actually were exon or were not exon. Specifically with the analysis of variants that affect exons, the number of variants in an interval is divided by the length of exons in the interval and then is multiplied by the length of the interval. With variants that do not affect exons, the number of variants in an interval is divided by the length of DNA that is not exon in the interval and then is multiplied by the length of the interval.

For phylogenetic analysis, we generated unrooted neighbor-joining trees^75^ by examining SNPs identified by Clair3 in Illumina data of 48 wild isolates sequenced mostly by CeDNR^19^. We made Newick tree files using many sizes of intervals, from 0.1 Mb to 2 Mb in 0.1 Mb increments and from 2 to 5 cM in 0.2 cM increments. VCF files were generated using a custom R script. Indexes of the VCF files and gunzip compressed versions of the VCF files were generated using BCFtools^76^. The resulting vcf.gz files were used to generate Newick files that examine the evolutionary relationship among the 40 wild isolates and the N2 reference genome for different genomic intervals using vcf-kit^77^, which generates an appropriate input file from the vcf.gz source files for MUSCLE (v3.8.31)^78^, which generates the Newick files. The R package ape (Analysis of Phylogenetics and Evolution)^79^ was used to extract cophenetic distances from Newick files. Phylogenetic trees were drawn with help of R packages ggplot2^80^, phyloseq^81^, grid^82^, gridExtra^83^, and ggmap^84^ using appropriate Newick files.

To identify distinct groups of DNA phylogenetically, we evaluated cophenetic distances recorded in Newick files. Two DNA with a cophenetic distance smaller than a predefined maximum cophenetic distance were declared as closely related to each other. Here, we picked 0.01 as the maximum cophenetic distance after evaluating cophenetic distances of 0.01–0.05 in 0.01 increments. In a given interval, there could be few to many different groups of DNA, and only one of these groups include the N2 reference. Notably within a genomic interval, one part could be closely related to the reference whereas another part could be divergent. To serve as a proofread, phylogenetic examination was made using genomic intervals of 0.1 Mb. For example, a result from an analysis using a ~ 1 Mb interval was compared against results from secondary analyses using ten or more 0.1 Mb intervals within the ~ 1 Mb interval. We accepted a DNA segment as being either closely related to or divergent from the reference genome only with the same conclusions from all analysis.

### Supplementary Information

Supplementary Information 1.

Supplementary Table 1.

## Data Availability

All sequencing data used in this manuscript has been made public by others. Materials, such as scripts used for data analysis, are available upon request (HYH).
