# Supplementary material for: Effect of recombination on genetic diversity of Caenorhabditis elegans
Source: Sci Rep. 2023 Sep 30;13:16425. doi: 10.1038/s41598-023-42600-5 (PMC10542817; doi:10.1038/s41598-023-42600-5)
Supplement: Supplementary file 1 — Supplementary Information 1. [file 41598_2023_42600_MOESM1_ESM.docx]

**Supplementary Text 1. Explanation of algebraic transformations for the mutation model**

**Equation 1.**

Three concepts (1), (2), and (3) explained in the main text are listed below.

(1) # variants by Sanger = DNA interval [Mb] * time * Sanger rate [variants Mb^-1^ time^-1^]

(2) # variants by Morgan = DNA interval [cM] * time * Morgan rate [variants cM^-1^ time^-1^]

(3) R coefficient [cM Mb^-1^] = Sanger rate / Morgan rate

We could redefine Sanger rate by multiplying the two sides of the equation (3) with Morgan rate. On the right side, Morgan rate divided by Morgan rate is one and thus could be removed.

(4) Morgan rate * R coefficient = Sanger rate / Morgan rate * Morgan rate

(5) Morgan rate * R coefficient = Sanger rate

With (5), we could revise (1) as follows, replacing the Sanger rate with the product of Morgan rate and the R coefficient.

(6) # variants by Sanger = interval [Mb] * time * Sanger rate

= interval [Mb] * time * Morgan rate * R coefficient

= interval (n) * time (d) * Morgan rate (M) * R coefficient (R)

= n * d * M * R

= R * n * d * M

With the right side of the equation (2), we both multiply by the interval in units of Mb and divide by the same interval in units of Mb. This is a simple trick, which does not change the value of the number of variants since we are dividing and multiplying using the same value (e.g. 10 * 5 = 10 / 2 * 2 * 5).

(7) # variants by Morgan = interval [cM] * time * Morgan rate

= interval [cM] / interval [Mb] * interval [Mb] * time * Morgan rate

Here in (7), interval [cM] / interval [Mb] is equal to interval [cM/Mb] or the recombination rate of the interval. The three intervals are identical, except that the same interval is measured three different ways, namely by Mb, cM, and cM/Mb.

(8) # variants by Morgan = interval [cM] / interval [Mb] * interval [Mb] * time * Morgan rate

= interval [cM/Mb] * interval [Mb] * time * Morgan rate

= interval (r) * interval (n) * time (d) * Morgan rate (M)

= r * n * d * M

The number of variants by Sanger (6) and Morgan (8) could be summed into (9). The sum of variants by Sanger and Morgan mechanisms is total variants. Notably, (6) and (8) share identical values (n * d * M), and thus the equation could be rewritten as (9). Equation (9) is equation 1 in the main text.

(9) # variants total = (R * n * d * M) + (r * n * d * M) = # by Sanger + # by Morgan

= (R + r) * n * M * d [equation 1]

**Equation 2.**

Starting with (8) and (7), we could determine the number of a specific variant subtype (e.g. indels), which is the product of all variants and the probability of a mutation being a specific mutation subtype. With Morgan mechanism (8), the modifier is the probability specific to Morgan mechanism (10). With Sanger mechanism (7), the modifier is the probability specific to Sanger mechanism (11). Importantly, (10) and (11) could be used for any variant subtype, such as transversions and indels of specific size.

(8) # variants by Morgan = r * n * d * M

(10) # indels by Morgan = r * n * d * M * probability of the variant being an indel (F_M_)

= F_M_ * r * n * d * M

(7) # variants by Sanger = R * n * d * M

(11) # indels by Sanger = R * n * d * M * probability of the variant being an indel (F_S_)

= F_s_ * R * n * d * M

The number of indels by Morgan (10) and Sanger (11) mechanisms could be summed into (12). Equation (12) is equation 2 in the main text.

(12) # indels total = (F_M_ * r * n * d * M) + (F_s_ * R * n * d * M)

= (F_M_ * r + F_S_ * R) * n * M * d [equation 2]

**Equation 3.**

Finally, (12) could be divided by (9) to describe the percentage of a specific mutation type (e.g. % indels) (13). Here, n * M * d divided by n * M * d on the right side is equal to one and thus could be removed. Equation (13) is equation 3 in the main text. Notably, nonlinear least squares (NLS) regression analysis could be performed without n * M * d, where M and d are unknown. This is useful because too many unknown values could cause the NLS analysis to fail (no convergence).

(12) # indels total = (F_M_ * r + F_S_ * R) * n * M * d

(9) # variants total = (R + r) * n * M * d

(13) % indels = ((F_M_ * r + F_S_ * R) * n * M * d) / ((R + r) * n * M * d)

= (F_M_ * r + F_S_ * R) / (R + r) [equation 3]

**Contribution by Morgan and Sanger mechanisms in mutation generation**

The number of variants by Morgan mechanism for a DNA interval (8) could be summed for all intervals (14). Same could be done with (7) into (15). Also, same with (9) into (16).

(8) # variants by Morgan for a DNA interval = r * n * d * M

(14) # variants by Morgan for all intervals = Σ (r * n * d * M)

(7) # variants by Sanger for a DNA interval = R * n * d * M

(15) # variants by Sanger for all intervals = Σ (R * n * d * M)

(9) # by Sanger + # by Morgan for a DNA interval = (R * n * d * M) + (r * n * d * M)

(16) # by Sanger + # by Morgan for all intervals = Σ (R * n * d * M) + Σ (r * n * d * M)

The proportional contribution of Morgan mechanism for all intervals or the whole genome is (14) divided by (16), which is (17).

(17) proportion by Morgan for the genome = Σ (r * n * d * M) / (Σ (R * n * d * M) + Σ (r * n * d * M))

(18) proportion by Sanger for the genome = Σ (R * n * d * M) / (Σ (R * n * d * M) + Σ (r * n * d * M))

With (17) and (18), time (d) and Morgan rate (M) are constant whereas the recombination rate of the interval (r) is not. Physical distance of all intervals (n) could be made identical, in which case (17) and (18) could be written as (19) and (20), respectively. With intervals of varying physical distance, (n) is different for different intervals, and we use (21) and (22) instead.

(19) proportion by Morgan for the genome = Σ r / (Σ R + Σ r)

(20) proportion by Sanger for the genome = Σ R / (Σ R + Σ r)

(21) proportion by Morgan for the genome = Σ (r * n) / (Σ (R * n) + Σ (r * n))

(22) proportion by Sanger for the genome = Σ (R * n) / (Σ (R * n) + Σ (r * n))

**Contribution by Morgan and Sanger mechanisms in mutation subtype generation**

This is very similar to the contribution calculations for all variants described above. Sum of (10) for the genome is (23). Sum of (11) for the genome is (24). Sum of (12) for the genome is (25).

(10) # indels by Morgan for a DNA interval = F_M_ * r * n * d * M

(11) # indels by Sanger for a DNA interval = F_s_ * R * n * d * M

(12) # indels total for a DNA interval = (F_M_ * r * n * d * M) + (F_s_ * R * n * d * M)

(23) # indels by Morgan for the genome = Σ (F_M_ * r * n * d * M)

(24) # indels by Sanger for the genome = Σ (F_S_ * R * n * d * M)

(25) # indels total for the genome = Σ (F_S_ * R * n * d * M) + Σ (F_M_ * r * n * d * M)

Next, (10) divided by (12) is (26). With identical physical distance for all intervals, (26) is equal to (27). With intervals of different physical distance, (26) is equal to (28). Finally, (11) divided by (12) is (29). With identical physical distance for all intervals, (29) is equal to (30). With intervals of different physical distance, (29) is equal to (31).

(26) proportion Morgan subtype = Σ (F_M_ * r * n * d * M) / (Σ (F_S_ * R * n * d * M) + Σ (F_M_ * r * n * d * M))

(27) proportion Morgan subtype = Σ (F_M_ * r) / (Σ (F_S_ * R) + Σ (F_M_ * r))

(28) proportion Morgan subtype = Σ (F_M_ * r * n) / (Σ (F_S_ * R * n) + Σ (F_M_ * r * n))

(29) proportion Sanger subtype = Σ (F_S_ * R * n * d * M) / (Σ (F_S_ * R * n * d * M) + Σ (F_M_ * r * n * d * M))

(30) proportion Sanger subtype = Σ (F_S_ * R) / (Σ (F_S_ * R) + Σ (F_M_ * r))

(31) proportion Sanger subtype = Σ (F_S_ * R * n) / (Σ (F_S_ * R * n) + Σ (F_M_ * r * n))

**With background selection and selective sweep**

To combine the mutation model with equations of background selection or selective sweep, we substituted the level of polymorphism in the absence of selection at linked sites (π_o_) with the total number of variants (R + r) * n * M * d in the mutation model.

Background selection with the mutation model

π = π_o_ * exp( -U / (s_d_ + r * (1 - F)) / (1 + F)) background selection alone

π = (R + r) * n * M * d * exp( -U / (s_d_ + r * (1 - F)) / (1 + F))

Selective sweep with the mutation model

π = π_o_ * r * (1 - F) / ((1 + F) * (r * (1 - F) + β)) selective sweep alone

π = (R + r) * n * M * d * r * (1 - F) / ((1 + F) * (r * (1 - F) + β))

All three models could be combined by nesting. For example, the combined equation for the mutation model and background selection could substitute π_o_ for selective sweep.

π = (R + r) * n * M * d * exp( -U / (s_d_ + r * (1 - F)) / (1 + F)) * r * (1 - F) / ((1 + F) * (r * (1 - F) + β))


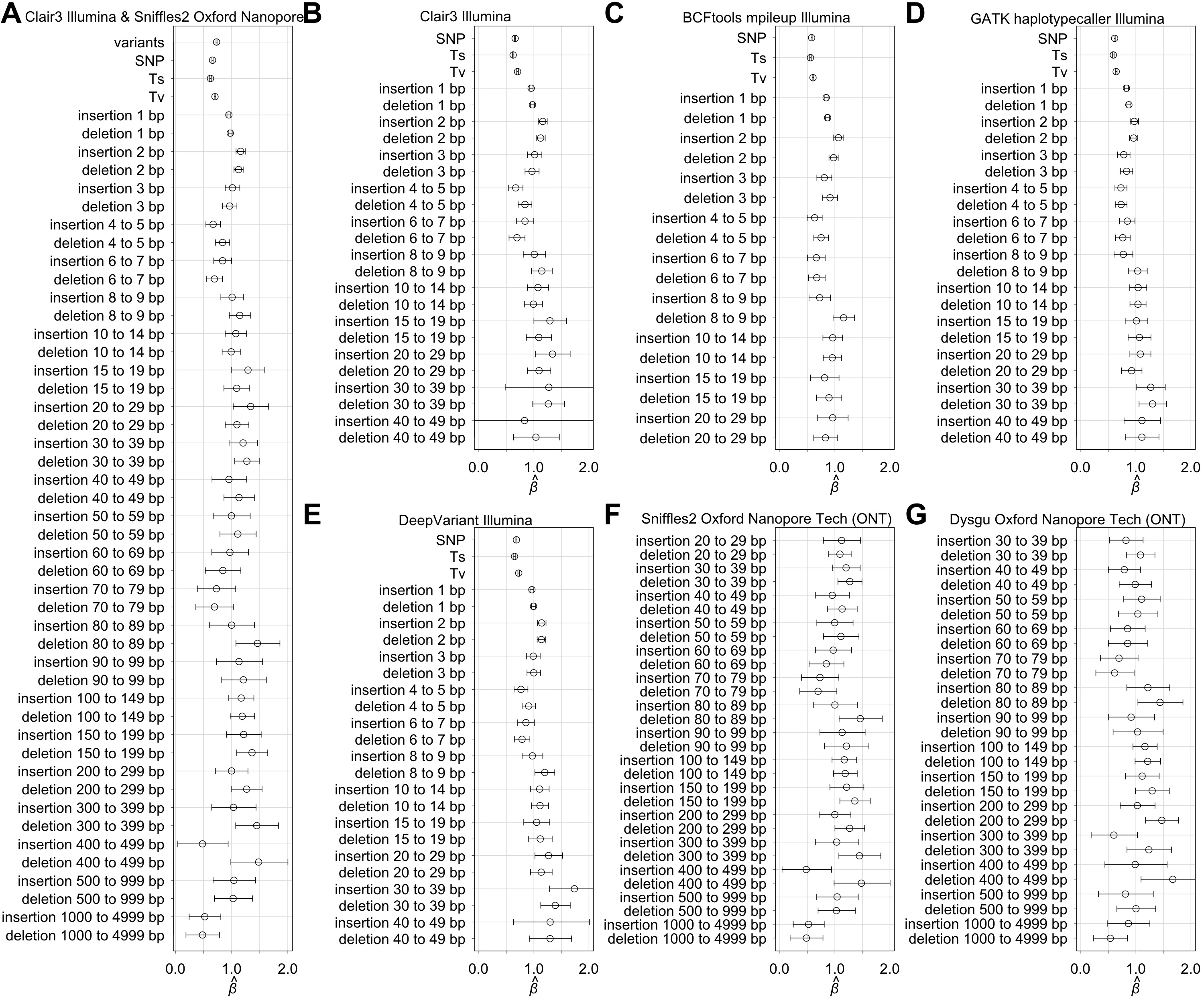


**Supplementary Figure 1.** **Recombination rate and variant abundance with CB4856.** Regression coefficient $\hat{\beta}$ and 95% confidence intervals examining the relationship between recombination rate and different variant subtype distributions in CB4856 HA are shown. Genomic intervals of ~0.6 Mb intervals are used. Variant data sets were obtained using (A) Clair3 with Illumina data for SNP and indels of up to 29 bp and Sniffles2 and ONT data for indels of 30 bp and larger, (B) Clair3 with Illumina data, (C) BCFtools with Illumina data, (D) GATK HaplotypeCaller with Illumina data, (E) DeepVariant with Illumina data, (F) Sniffles2 with ONT data, (G) Dysgu with ONT data.


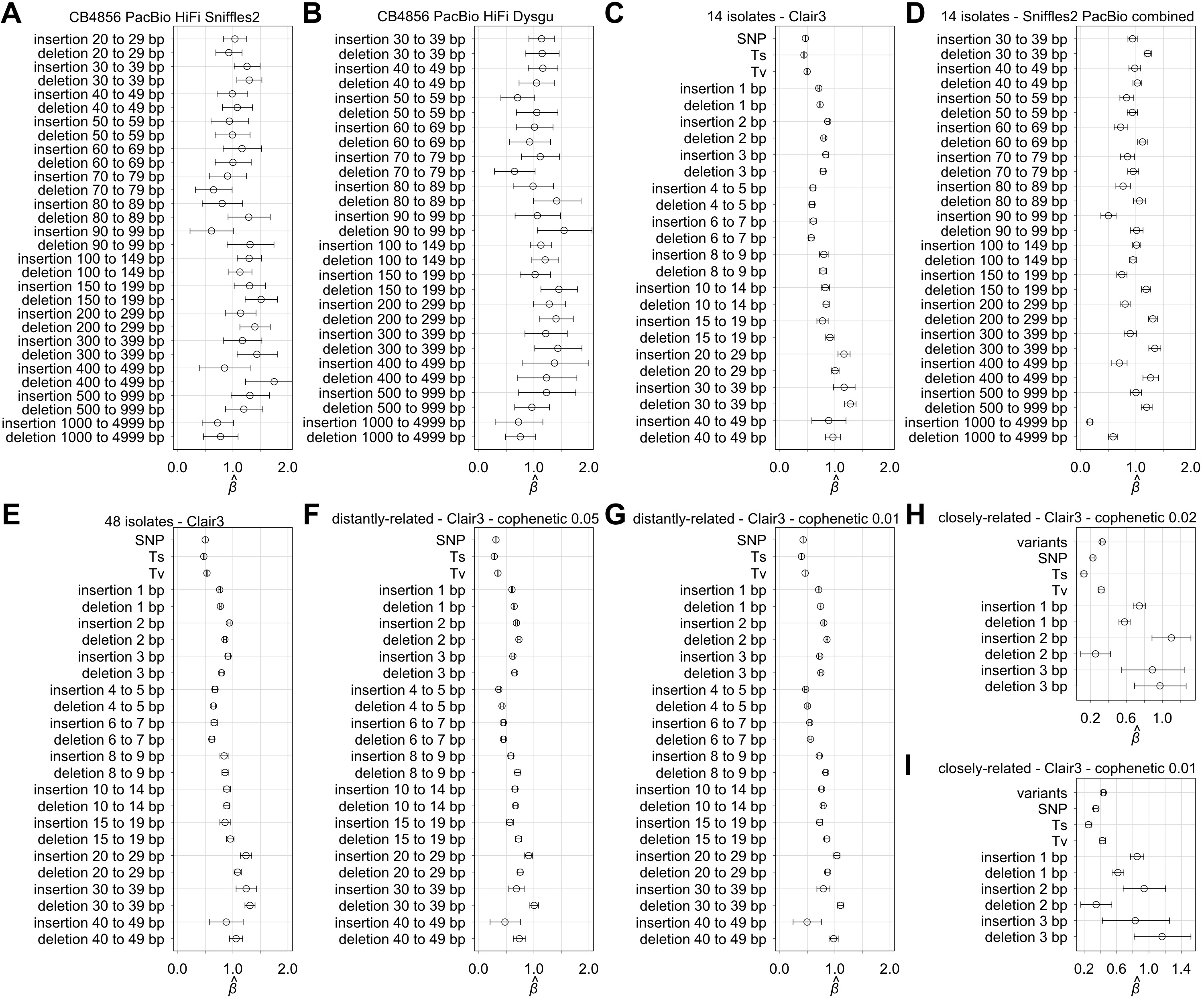


**Supplementary Figure 2.** **Recombination rate and variant abundance in wild isolates with and without PacBio data.** Regression coefficient $\hat{\beta}$ and 95% confidence intervals examining the relationship between recombination rate and different variant subtype distributions in various sets of wild isolates are shown. Genomic intervals of ~0.6 Mb intervals are used. Variant data sets were obtained using (A) Sniffles2 with PacBio HiFi data of CB4856 HA, (B) Dysgu with PacBio HiFi data of CB4856 HA, (C) Clair3 with Illumina data of 14 wild isolates, which also have PacBio data, (D) Sniffles2 with first-generation PacBio data of 14 wild isolates, (E) Clair3 with Illumina data of 48 wild isolates, (F) Clair3 with divergent DNA by maximum cophenetic distance of 0.05 using 48 wild isolates with Illumina data, (G) Clair3 with divergent DNA by maximum cophenetic distance of 0.01 using 48 wild isolates with Illumina data, (H) Clair3 with closely-related DNA by maximum cophenetic distance of 0.02 using 48 wild isolates with Illumina data, and (I) Clair3 with closely-related DNA by maximum cophenetic distance of 0.01 using 48 wild isolates with Illumina data.


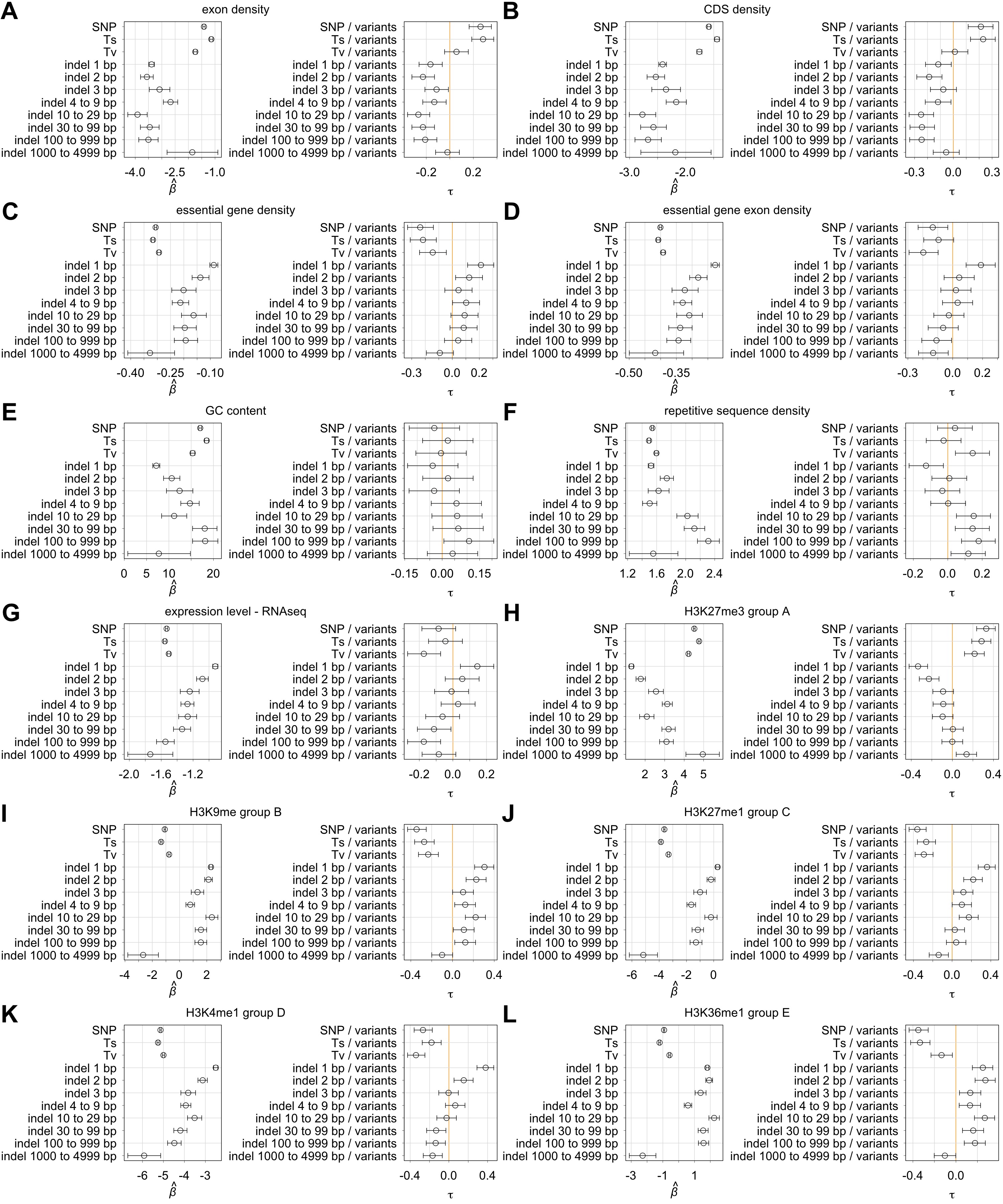


**Supplementary Figure 3. Other covariates and variant subtype abundance and proportions.** Regression coefficient $\hat{\beta}$ (left), Kendall tau values (right), and 95% confidence intervals examining the relationship between different covariates and various variant subtype distributions and proportions are shown. Variants in CB4856 and genomic intervals of ~0.6 Mb intervals are used. Covariates are (A) exon density, (B) coding DNA sequence density, (C) essential gene density, (D), essential gene exon density, (E) GC content, (F) repetitive sequence density, (G) expression level according to RNAseq, and (H-L) average methylation states.

**
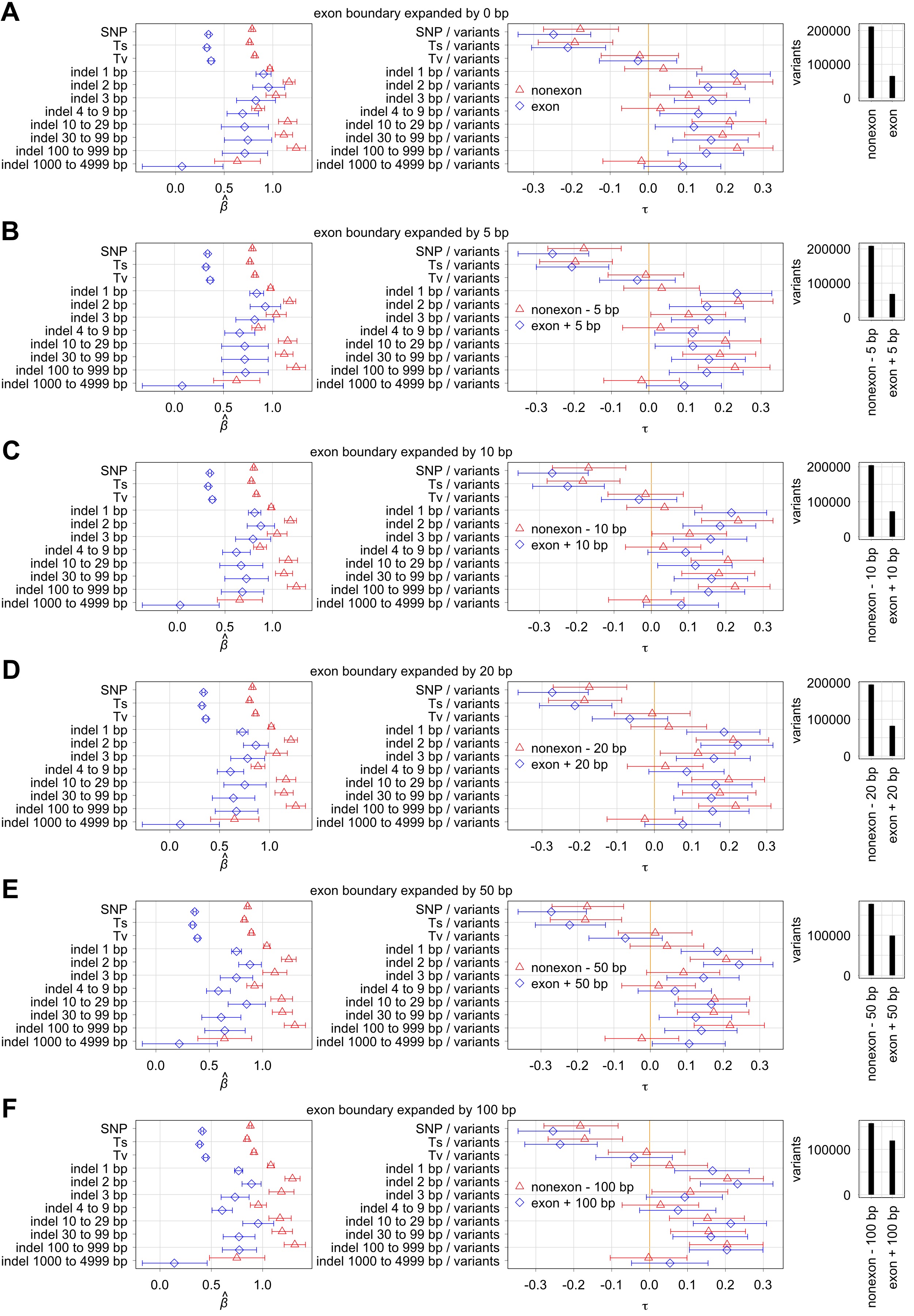
Supplementary Figure 4. Effect of expanding exon boundaries.** Regression coefficient $\hat{\beta}$ (left), Kendall tau (middle) and 95% confidence interval between recombination rate and variant subtype abundance and between recombination rate and variant subtype proportion are shown. Total number of variants that affect exon or do not affect exon are shown on right. Variants in CB4856 and genomic intervals of ~0.6 Mb intervals are used. Exon boundaries are expanded by (A) 0 bp, (B) 5 bp, (C) 10 bp, (D) 20 bp, (E) 50 bp, or (F) 100 bp.


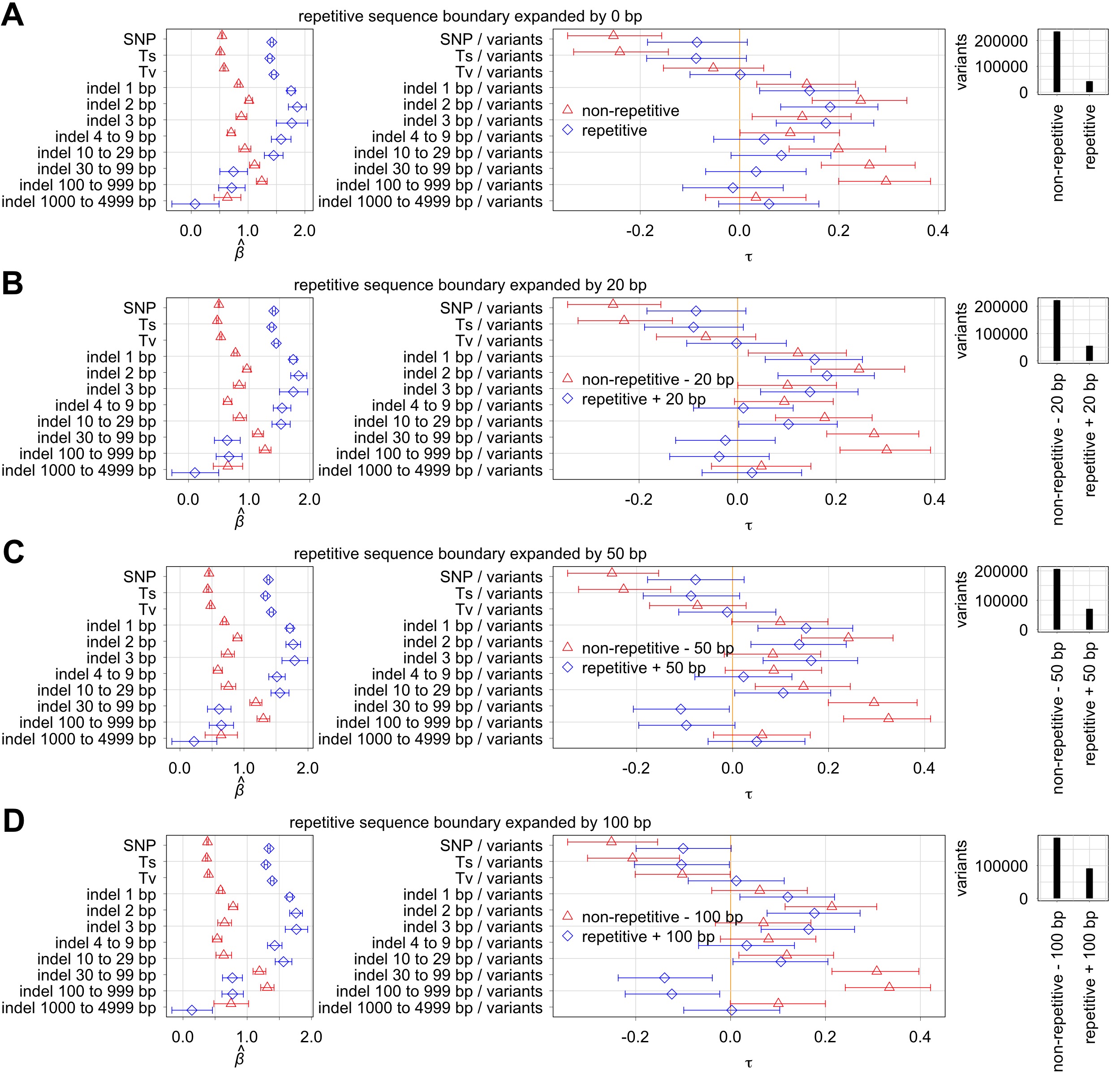


**Supplementary Figure 5. Effect of expanding repetitive sequence boundaries.** Regression coefficient $\hat{\beta}$ (left), Kendall tau (middle) and 95% confidence interval between recombination rate and various variant subtype abundance and proportion are shown. Total number of variants that affect repetitive sequence or do not affect repetitive sequences are shown. Variants in CB4856 and genomic intervals of ~0.6 Mb intervals are used. Repetitive sequence boundaries are expanded by (A) 0 bp, (B) 20 bp, (C) 50 bp, and (D) 100 bp.


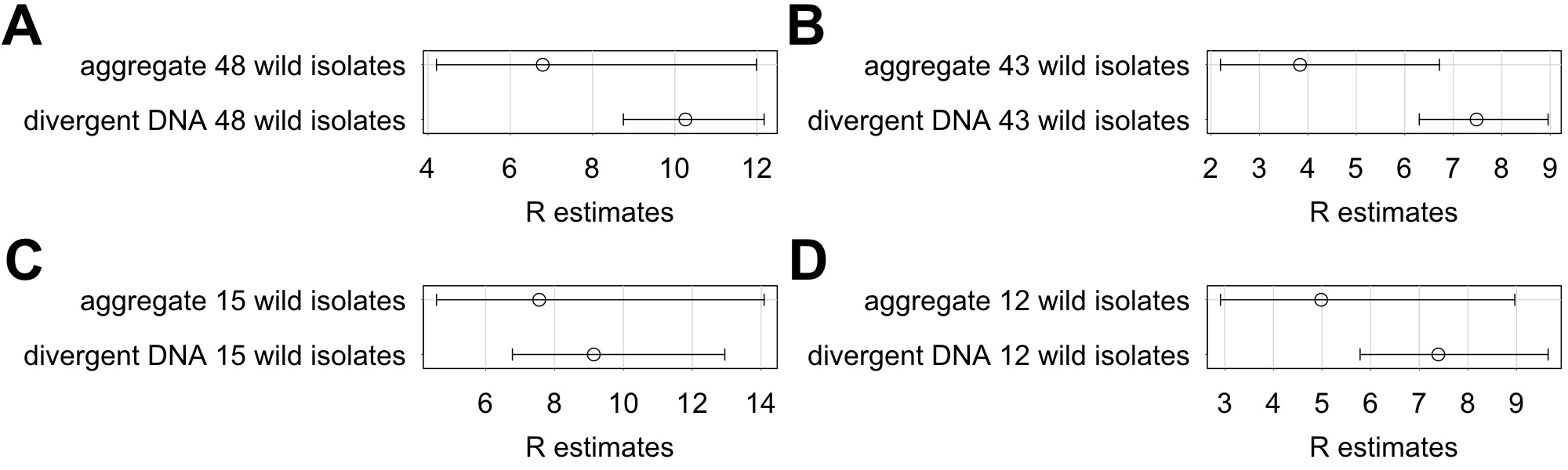


**Supplementary Figure 6. Effect of phylogenetic examination on R estimates.** R estimates and 95% confidence intervals are shown using variant data from different sets of wild isolates with and without phylogenetic examination. Aggregate data without phylogenetic examination, divergent DNA data from phylogenetic examination, and genomic intervals of ~0.6 Mb intervals are used. Variant data sets are of (A) 48 wild isolates, (B) 43 wild isolates without the outlier trio and JU2526 and JU775, (C) 15 wild isolates with PacBio data, and (D) 12 wild isolates with PacBio data and excluding the outlier trio. JU2526 and JU775 have large numbers of variants similar to those of the outlier trio in the middle of chromosomes IV, V, and X (see Figure 2D, 2F, and 3C).


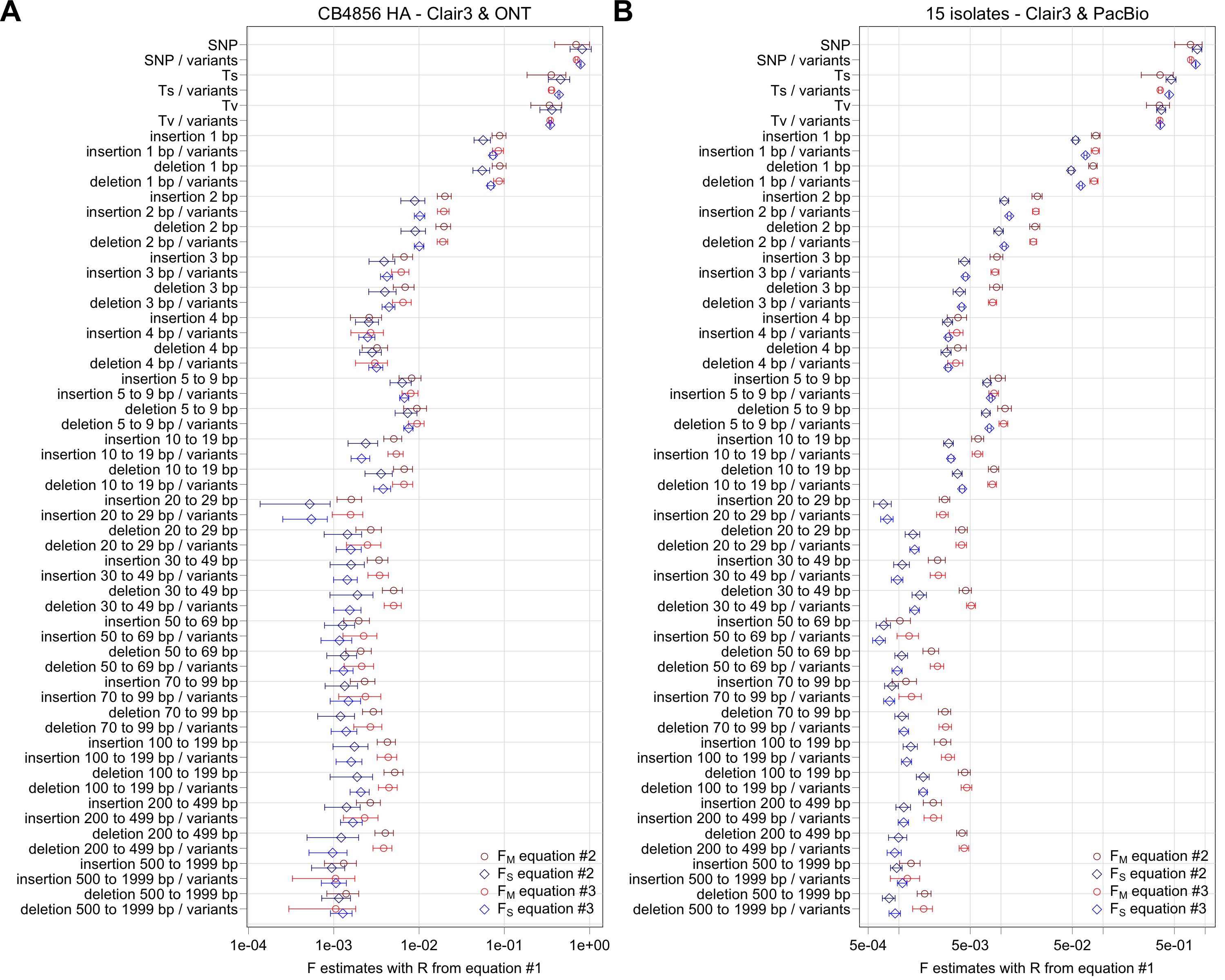


**Supplementary Figure 7. Comparison of F_M_ and F_S_ estimates using equations #2 and #3 with R from equation #1.** F_M_ and F_S_ estimates and 95% confidence intervals from examining variant subtypes including SNP, Ts, Tv, and insertions and deletions of various size ranges and their corresponding variant subtype proportions. Genomic intervals of ~0.6 Mb are used with variant data of (A) CB4856 with ONT and Illumina data and (B) divergent DNA of 15 wild isolates with PacBio and Illumina data.

**
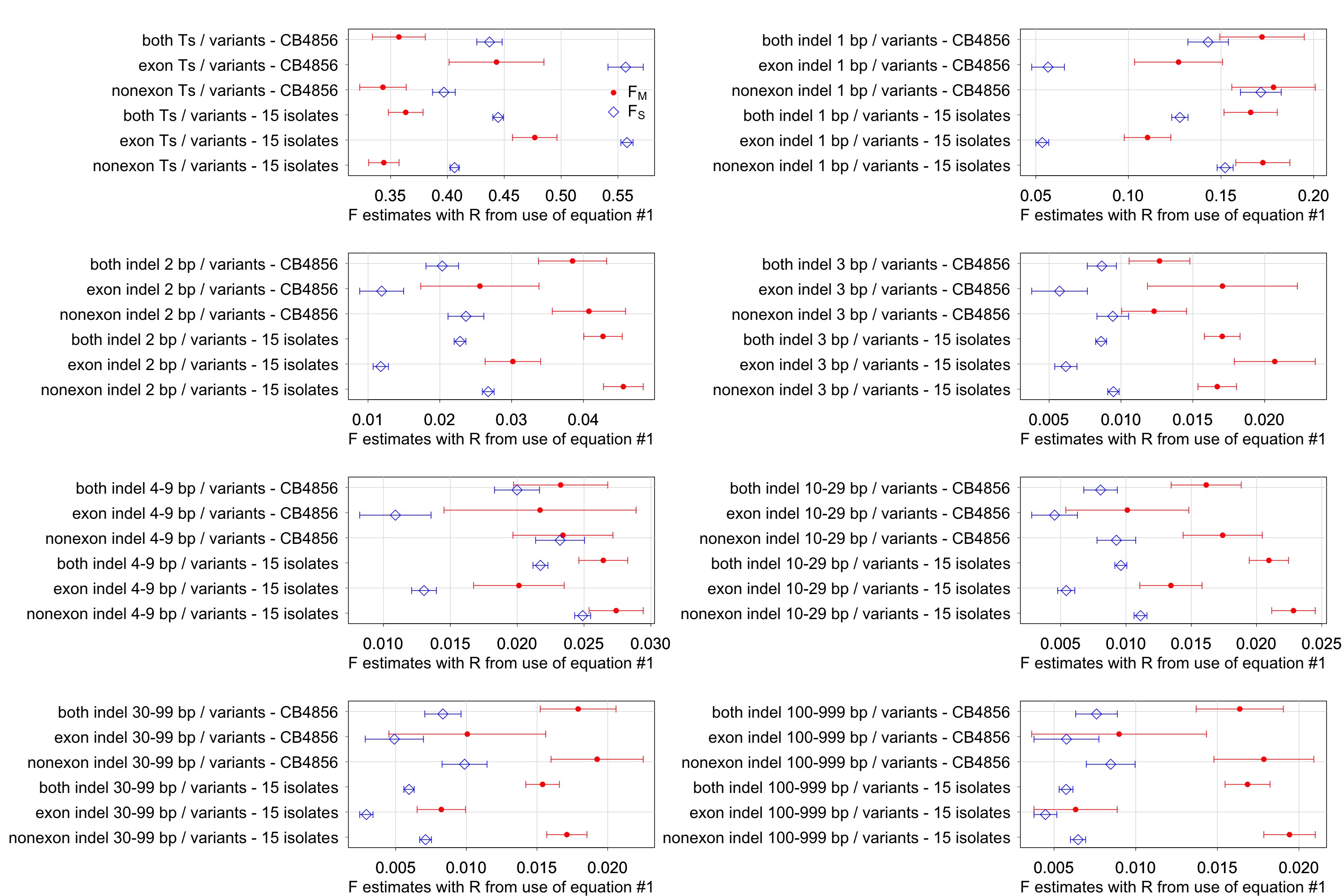
**

**Supplementary Figure 8. F_M_ and F_S_ estimates with variants that affect or do not affect exons.** F_M_ and F_S_ estimates and 95% confidence intervals from examining various variant subtype proportions are shown. Separate estimates are shown using variants that do not affect exons, variants that affect exons, and both type of variants. Variant data of CB4856 with ONT and Illumina data, variant data of divergent DNA of 15 wild isolates with PacBio and Illumina data, and genomic intervals of ~0.6 Mb are used.


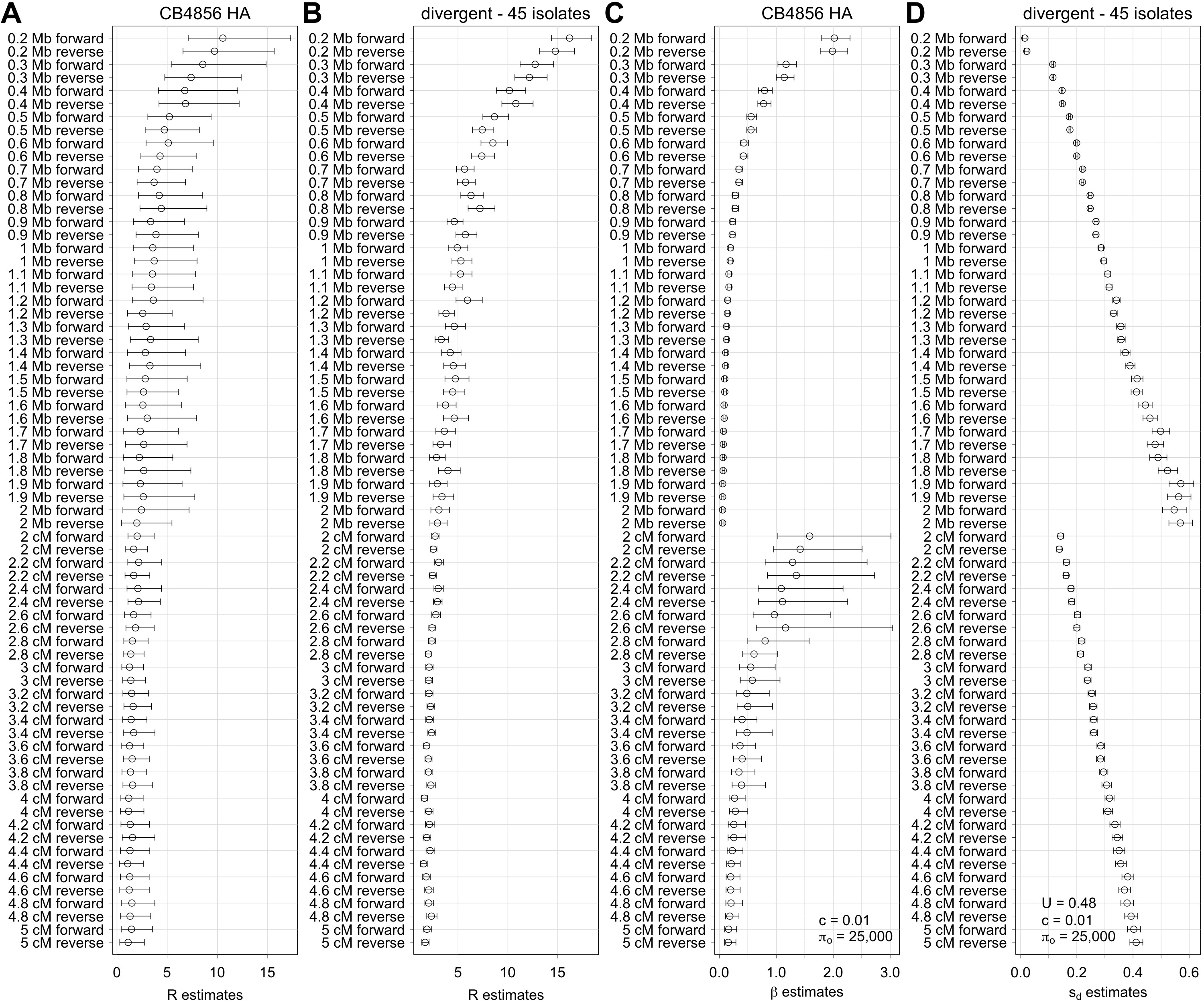


**Supplementary Figure 9. Interval size effect on variant abundance analysis.** (A-B) R estimates and 95% confidence intervals using (A) CB4856 with ONT and Illumina data and (B) divergent DNA of 45 wild isolates from phylogenetic examination. (C) β estimates and 95% confidence intervals using selective sweep equation and CB4856 data. Presumed c and π_0_ values are 0.01 and 25,000, respectively. (D) Estimates of s_d_ and 95% confidence intervals using background selection equation and divergent DNA of 45 wild isolates from phylogenetic examination. Presumed U, c, and π_0_ values are 0.48, 0.01, and 25,000, respectively.


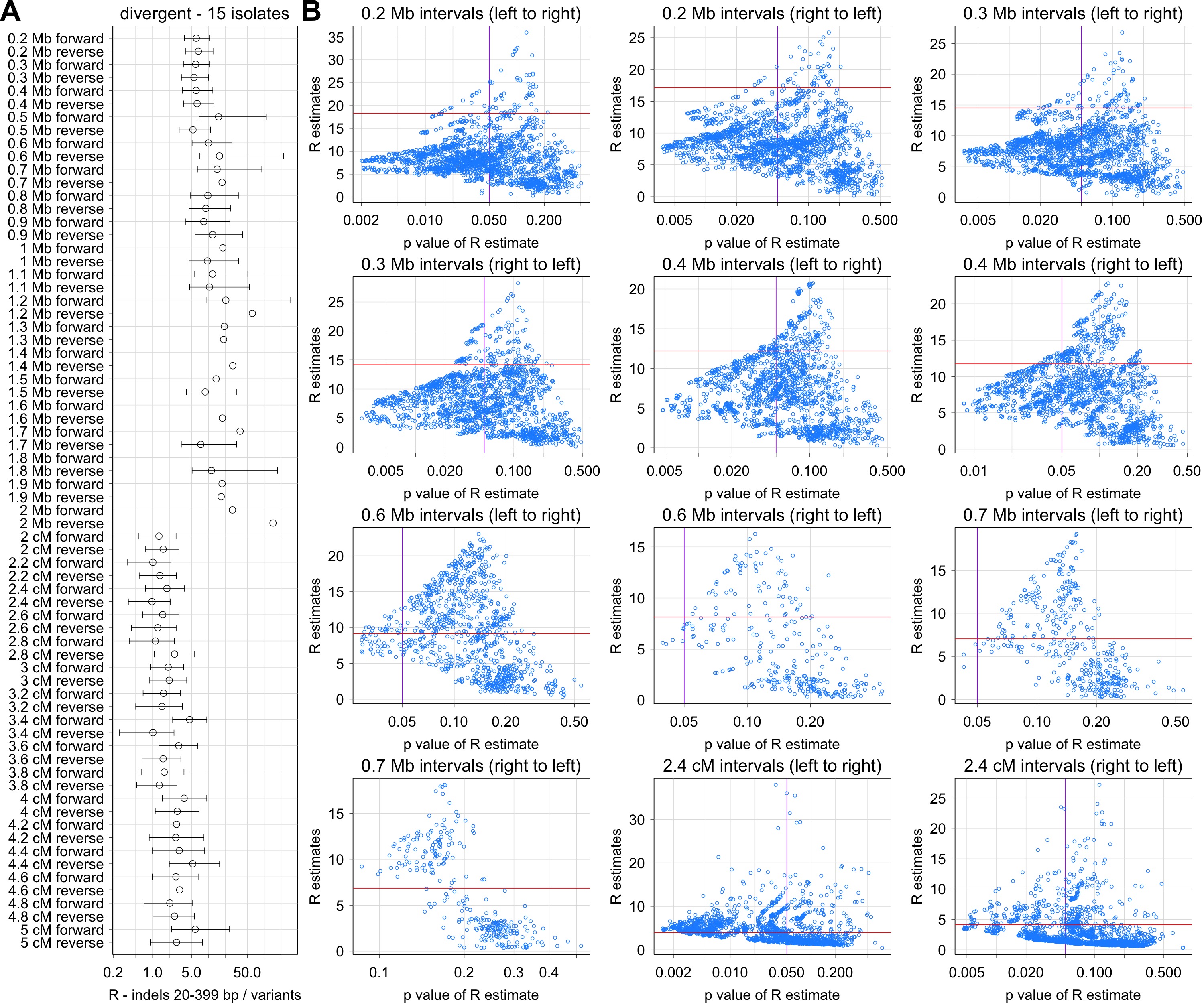


**Supplementary Figure 10. Interval size effect on variant subtype proportion analysis.** (A) R estimates and 95% confidence intervals examining indels of 20 to 399 bp out of variants using divergent DNA of 15 wild isolates with PacBio and Illumina data. (B) R estimates and corresponding p values with NLS analysis of variant subtype proportions using equation #3 alone. Different interval sizes (e.g. 0.2 Mb, 0.3, Mb, 0.6 Mb, 2.4 cM) and orientation (forward or left to right and reverse or right to left) were used. Estimates from poor NLS analysis results as defined by inability to calculate 95% confidence intervals for R, F_M_, and F_S_ were censored from 2586 variant subtype proportions examined. Small number of R estimates below zero wee also censored. Purple vertical line marks the p value of 0.05. Red horizontal line indicates R estimate obtained with equation #1.


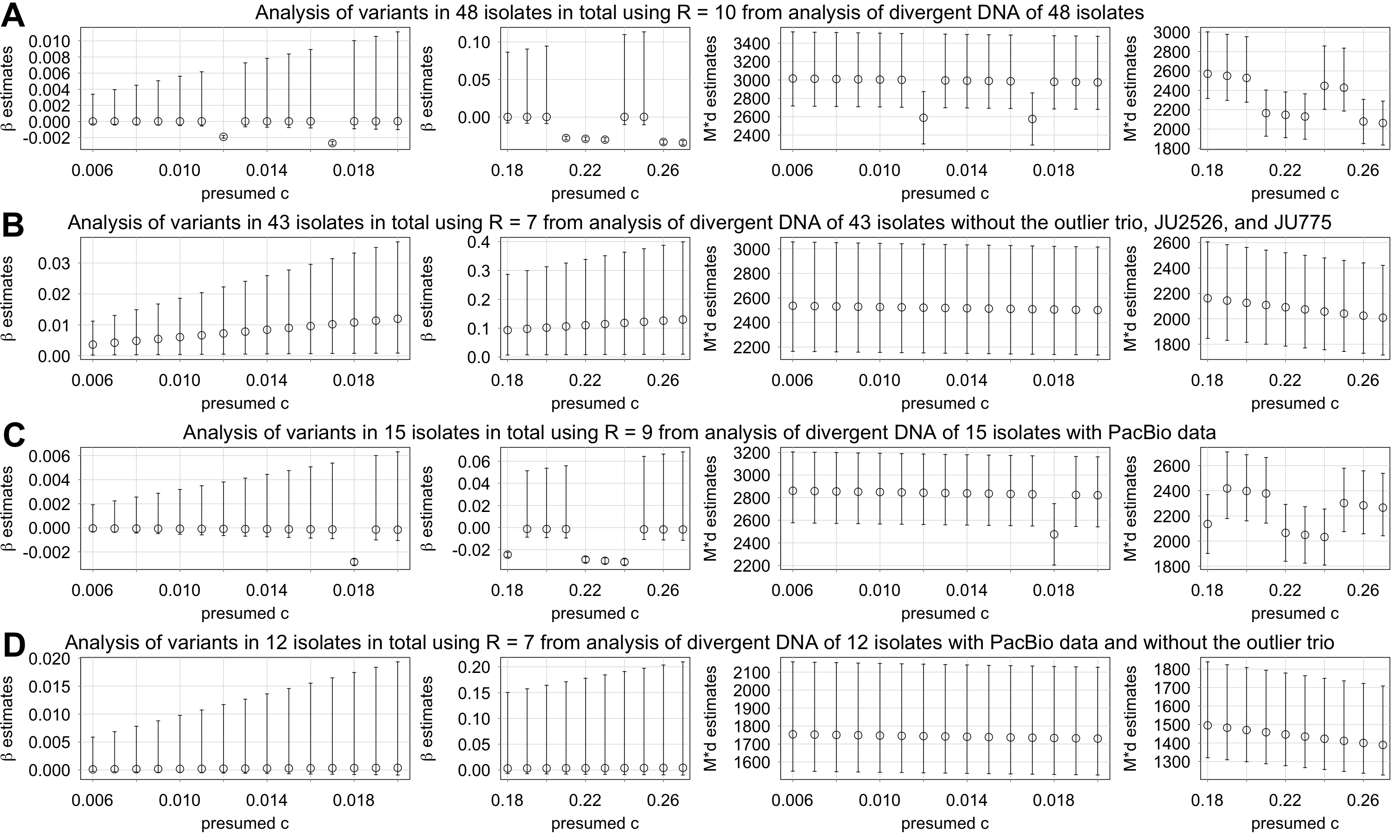


**Supplementary Figure 11. Evaluation of β combining the mutation model with selective sweep with different variant data sets.** Estimates of β (left 2) and M*d (right 2) with 95% confidence intervals using aggregate variant data sets of (A) 48 wild isolates, (B) 43 wild isolates excluding the outlier trio and JU2526 and JU775, (C) 15 wild isolates with PacBio and Illumina data, and (D) 12 wild isolates with PacBio and Illumina data and excluding the outlier trio. Genomic intervals of ~0.6 Mb are used.


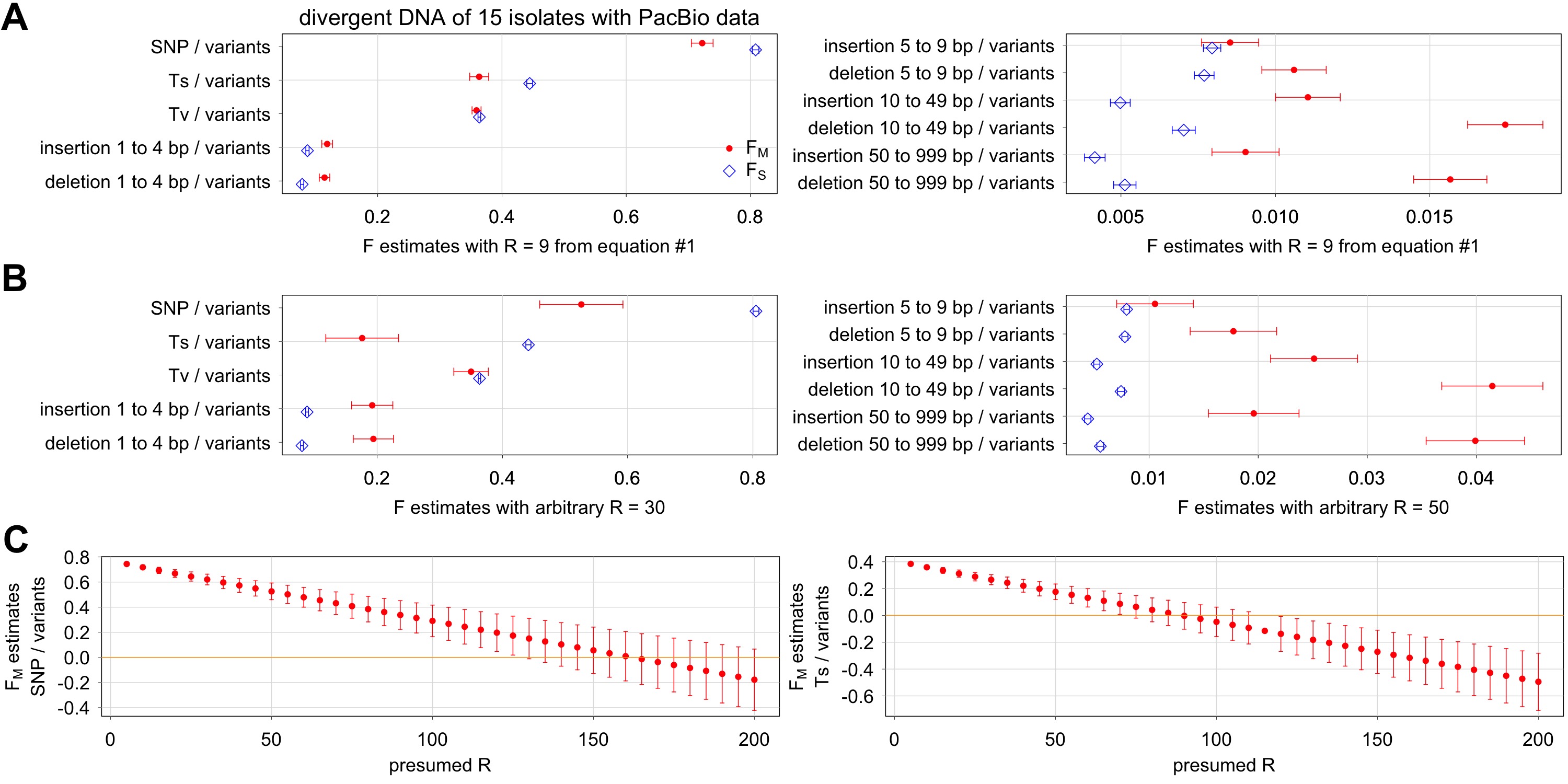


**Supplementary Figure 12. Upper limit of R value that could explain the correlation between recombination rate and all variant subtype proportions.** F_M_ and F_S_ estimates and 95% confidence intervals are shown for variant subtype proportions including SNP out of variants, Ts out of variants, Tv out of variants, and insertions and deletions of various size ranges out of variants. Variant data od divergent DNA of 15 wild isolates with PacBio and Illumina data and genomic intervals of ~0.6 Mb are used. (A) Estimates with R value calculated using equation #1. (B) Estimates with arbitrary R value of 100. Orange line at F value of 0 indicates lower limit of realistic F_M_ and F_S_ values. (C) Estimates of F_M_ values of SNP out of variants (left) and Ts out of variants (right) with a series of arbitrary R values.

**
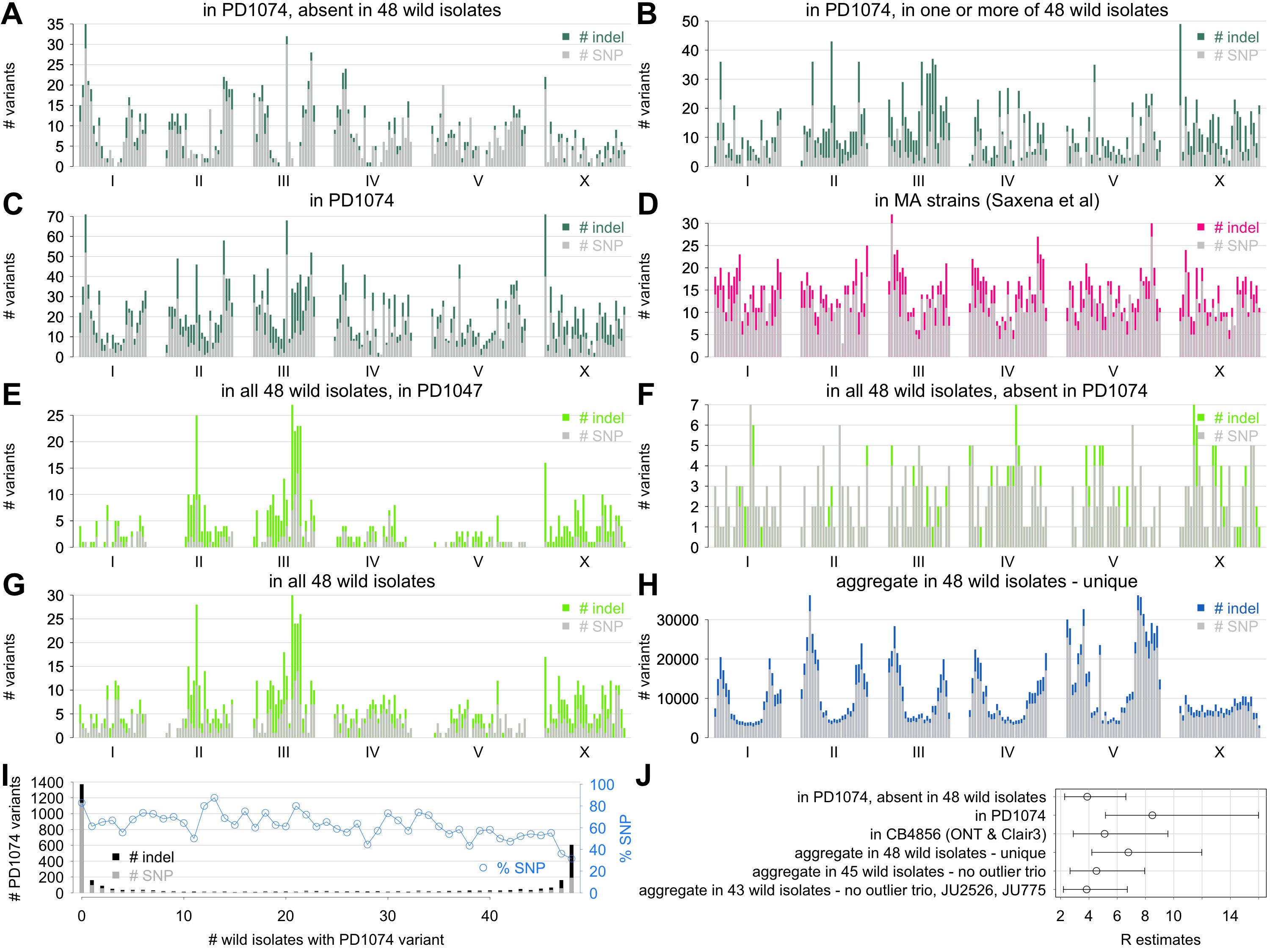
Supplementary Figure 13. Variant abundance in PD1074 and among variants that are present in all 48 wild isolates.** (A-H) Number of variants in ~0.6 Mb intervals across six chromosomes are shown. SNP is indicated by gray bar, and indel by colored bar. (A) PD1074 variants that are absent in the 48 isolates. (B) PD1074 variants that are present in one or more of the 48 isolates. (C) PD1074 variants in total. (D) Variants in Saxena et. al. (2019) MA strains. (E) Variants that are present in all 48 isolates and in PD1074. (F) Variants that are present in all 48 isolates but not in PD1074. (G) Variants that are present in all 48 isolates in total. (H) Variants in 48 wild isolates in aggregate. (I) Number of PD1074 variants are shown as a bar graph, grouped by the number of wild isolates among the 48 wild isolates, in which the PD1074 variant is also present. SNP is indicated by gray bar, and indel by black bar. Also, percentages of variants that are SNP are shown as a line graph with blue circles. (J) R estimates and 95% confidence intervals using data sets of PD1074 variants that are not in any of the 48 isolates, PD1074 variants in total, CB4856 variants (including ONT data), variants in aggregate in the 48 isolates, variants in aggregate in 45 wild isolates excluding the outlier trio (ECA36, ECA396, XZ1516), and variants in aggregate in 43 wild isolates excluding the outlier trio, JU2526, and JU775. All variant data are from Clair3 variant calling only except as indicated with CB4856 in (J).
